# Importance of the Cyclic Cystine Knot Structural Motif for Immunosuppressive Effects of Cyclotides

**DOI:** 10.1021/acschembio.1c00524

**Published:** 2021-09-30

**Authors:** Roland Hellinger, Edin Muratspahić, Seema Devi, Johannes Koehbach, Mina Vasileva, Peta J. Harvey, David J. Craik, Carsten Gründemann, Christian W. Gruber

**Affiliations:** # Center for Physiology and Pharmacology, Medical University of Vienna, Schwarzspanierstr. 17, Vienna 1090, Austria; § Institute for Molecular Bioscience, Australian Research Council Centre of Excellence for Innovations in Peptide and Protein Science, The University of Queensland, Brisbane, Queensland 4072, Australia; $ Institute for Infection Prevention and Hospital Epidemiology, Center for Complementary Medicine, Faculty of Medicine, University of Freiburg, Breisacher Str. 115B, Freiburg 79106, Germany; & Translational Complementary Medicine, Department of Pharmaceutical Sciences, University of Basel, Klingelbergstr. 80, Basel 4056, Switzerland

## Abstract

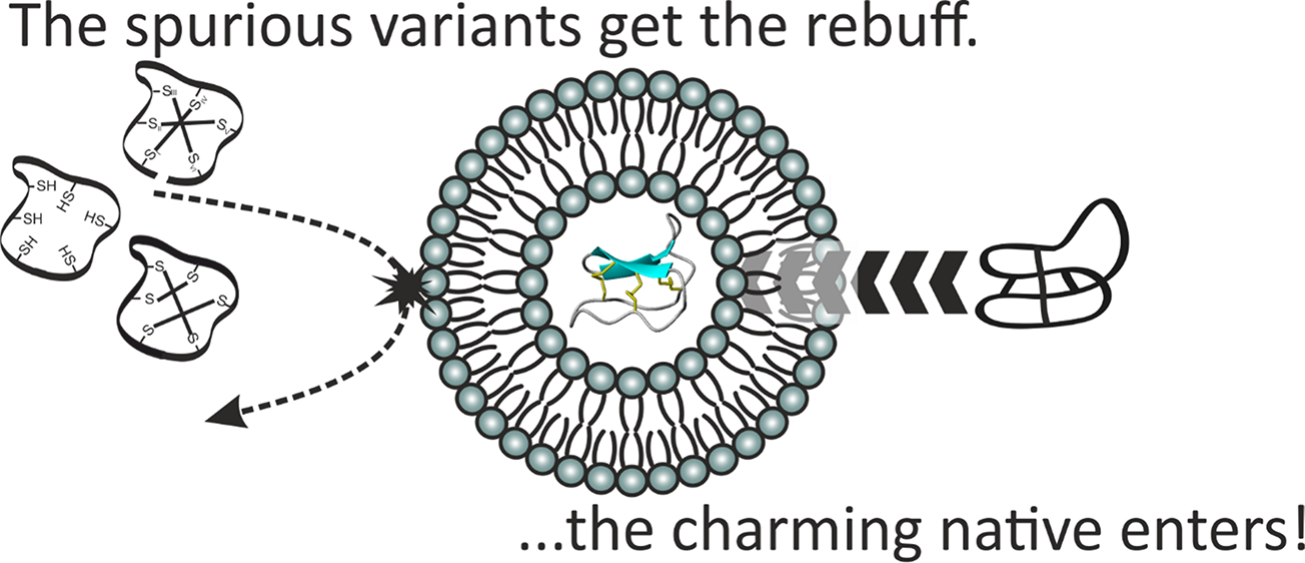

The cyclotide T20K inhibits the proliferation of human immune cells and is currently in clinical trials for multiple sclerosis. Here, we provide novel functional data and mechanistic insights into structure–activity relationships of T20K. Analogs with partial or complete reduction of the cystine knot had loss of function in proliferation experiments. Similarly, an acyclic analog of T20K was inactive in lymphocyte bioassays. The lack of activity of non-native peptide analogs appears to be associated with the ability of cyclotides to interact with and penetrate cell membranes, since cellular uptake studies demonstrated fast fractional transfer only of the native peptide into the cytosol of human immune cells. Therefore, structural differences between cyclic and linear native folded peptides were investigated by NMR to elucidate structure–activity relationships. Acyclic T20K had a less rigid backbone and considerable structural changes in loops 1 and 6 compared to the native cyclic T20K, supporting the idea that the cyclic cystine knot motif is a unique bioactive scaffold. This study provides evidence that this structural motif in cyclotides governs bioactivity, interactions with and transport across biological membranes, and the structural integrity of these peptides. These observations could be useful to understand the structure–activity of other cystine knot proteins due to the structural conservation of the cystine knot motif across evolution and to provide guidance for the design of novel cyclic cysteine-stabilized molecules.

## Introduction

Disulfide bonds formed between the side chains of cysteine residues during oxidative folding define the molecular architecture and, accordingly, the biological properties of many peptides and proteins. Nature has evolved a variety of structural motifs stabilized by one or more disulfide bonds.^1^ A standout example is the cystine knot motif, which occurs in all domains of life and has essential biological roles, for instance, in human peptide hormones^2,3^ or in spider toxins for prey hunting.^4^ This structural motif is defined by three intertwined disulfide bonds, two of which form a ring that is penetrated by the third disulfide bond. All 15 possible connectivities of three disulfide bonds are known to occur in nature^3^ but the I-IV, II-V, and III-VI connectivities, which emerge in the growth factor, inhibitor, and cyclic cystine knots, respectively, are the most common.^5^ In growth factor cystine knots, the penetrating disulfide bond is formed between cystines I and IV, whereas it is between III and VI in the inhibitor cystine knots and cyclic cystine knots.^6,7^

Cyclotides are the only family of proteins containing the cyclic cystine knot.^5^ They are widely distributed throughout flowering plants and have been isolated in species of the Rubiaceae, Violaceae, Cucurbitaceae, Fabaceae, Solanaceae, and Poaceae.^8^ A single plant species can express >160 distinct cyclotides,^9^ and their number is estimated to exceed 150,000 family members.^9,10^ Cyclotide-bearing plants are well established in traditional medicine,^11−14^ and it is not surprising that cyclotides have been studied for various bioactive properties.^8,15−17^ A key discovery for the application of cyclotides as therapeutic leads has been the elucidation of their immunosuppressive properties.^18^ Indeed, a single amino acid mutant of the prototypic cyclotide kalata B1, i.e., [T20K]-kalata B1 (termed “T20K”), is currently under clinical development as a drug candidate for multiple sclerosis.^19,20^ T20K has been extensively studied for its immune cell modulatory activity,^11,18,19,21,22^ and it affects proliferation of human and mouse T-lymphocytes via concentration-dependent and reversible inhibition of interleukin-2 (IL2) cytokine signaling.

**Figure 1:**
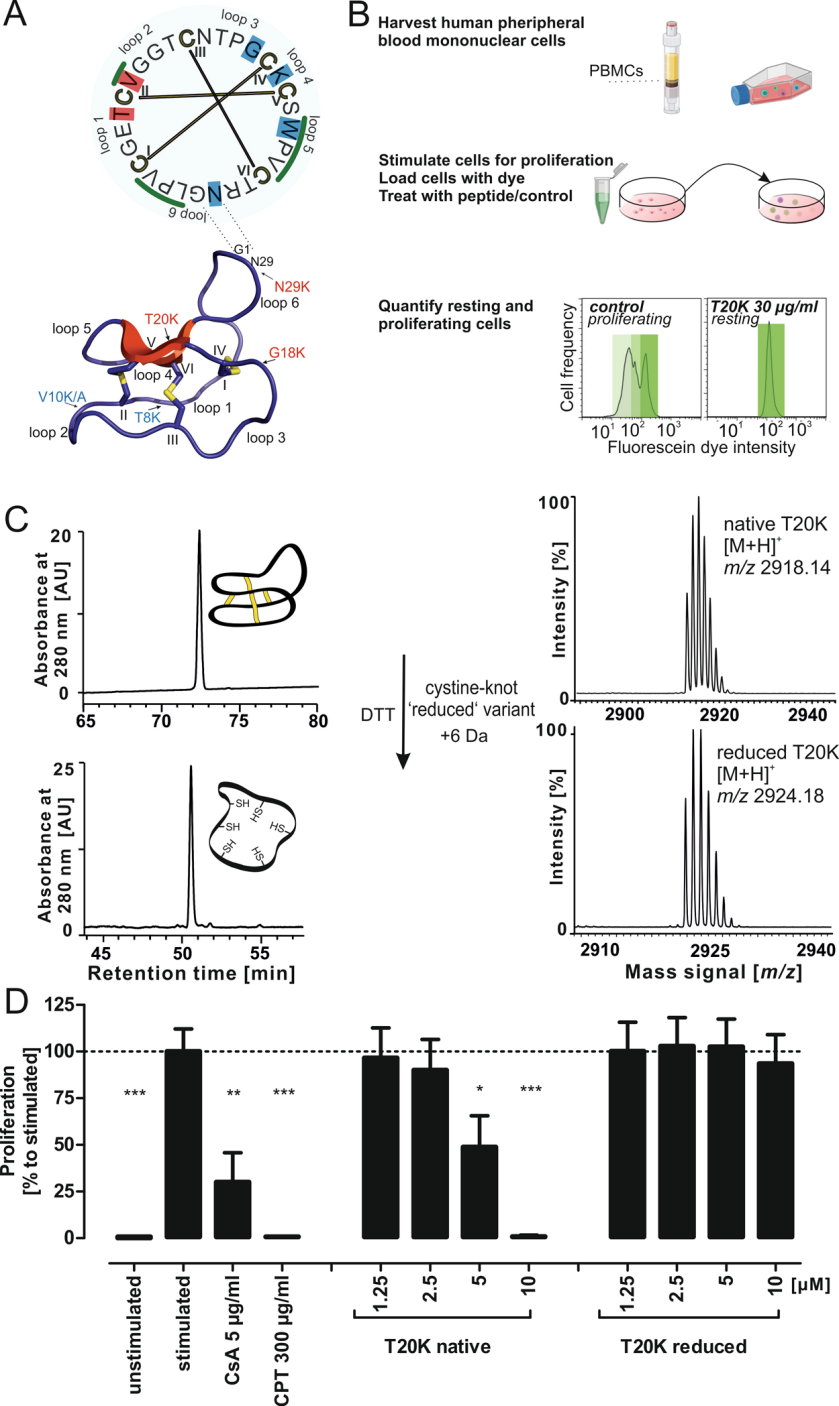
Structure of the cyclotide T20K and antiproliferative activity of native vs reduced peptide. (A) A schematic illustration of the head-to-tail cyclized peptide T20K is provided. A cyclic sequence is shown on the top, the disulfide connectivity is indicated in yellow bars, and bold Roman letter numbers the cysteines from the first one in the originating gene. The intercysteine loops are also labeled beginning from the native N-terminus. Previous mutational studies are highlighted (amino acid with background colors): red indicates loss of function residues and blue indicates residues amenable for mutagenesis without changing the bioactivity of the peptide. Residues identified to contribute to the hydrophobic patch of T20K are indicated with a green overscore. The 3D structure of T20K is modeled in a cartoon form. β-sheet structures are shown in red, and cystines are shown in yellow color. The intercystine loops and cysteine residue numbering are equivalent to the circular illustration. The residues studied via amino acid mutagenesis are indicated with an arrow (red shows active and blue color assigns inactive mutants). (B) A schematic illustration of the proliferation assay is shown to the right. The isolated naïve cells become activated for proliferation by the T-cell receptor-like stimulation with anti-CD3 and anti-CD28 antibodies. The PBMCs are loaded with the CFDA-SE dye to track proliferation. The stimulated PBMCs are treated with various concentrations of peptide and with control compounds. The population of resting or proliferating cells is analyzed using fluorescence-assisted cell sorting. The prototypic peptide T20K induces a strong antiproliferative effect similar to CsA (cyclosporine A). This figure was created with BioRender.com. (C) Synthetic native folded T20K (*m*/*z* 2918.14) was treated with dithiothreitol to yield a fully reduced peptide variant (*m*/*z* 2923.18). (D) Native T20K revealed a dose-dependent antiproliferative effect, whereas the cystine knot reduced variant lost activity in the tested concentration range up to 10 μM (∼29 μg/mL). All data represent mean ± standard deviation of three biological replicates, expressed relative to stimulated control with added PBS (=100%); CsA is a positive control for an immunosuppressant, and camptothecin (CPT) is a control for an antiproliferative and apoptosis-inducing compound. Asterisks (*p < 0.05, **p < 0.01, ***p < 0.001) indicate significant differences compared to stimulated control.

In the experimental autoimmune encephalitic (EAE) mouse model of multiple sclerosis, T20K modulates Th_1_ and Th_17_ cell function in diseased animals and significantly delays disease progression and reduces its severity.^19^ The peptide is orally active in this model, which is considered a “holy grail” in peptide drug development, suggesting that the cyclic cystine knot structure of T20K is important for stability, since it confers protection against proteolytic and acidic degradation.^23,24^ Mutational studies for the antiproliferative effects of T20K suggest that Thr^8^ and Val^10^ (loop 1 or 2, respectively; Figure 1A) are essential for bioactivity, since a Lys or Ala replacement at these positions leads to a loss of function. On the other hand, Ala/Lys mutants of residues G18, T20, or N29 (loops 3, 4, or 6, respectively; Figure 1A) do not affect antiproliferative activity.^21^ Similarly, bioactivity was unaffected when replacing W23 with the non-proteinogenic amino acid para-benzophenylalanine.^22^ An all D-amino acid peptide analogue of T20K has reduced bioactivity, which might be due to an unexplored role of a molecular receptor in T20K’s mode of action.^21^ Overall, these studies suggest that a surface-exposed hydrophobic patch in T20K (L2, P3, V4, V10, W23, P24, and V25; Figure 1A) determines its immunosuppressive activity.^25,26^

Cyclotides have attracted attention as ultrastable naturally occurring mini proteins.^8,15,16^ Their cyclic cystine knot (CCK) motif tolerates intercysteine sequence variation, while the structural integrity of the cystine knot remains unaffected.^27^ This structural plasticity may be exploited in drug design applications for the development of peptide pharmaceuticals.^28^ In fact, many bioactive sequences have been successfully grafted onto the CCK scaffold, which has resulted in novel peptides modulating, for instance, cell surface receptors (e.g., the melanocortin 4- or the bradykinin B1 receptor),^29,30^ the blood coagulation protein factor XIIa,^31^ and intracellular enzymes (e.g., tumor suppressor protein p53 or tumor activator SET).^32,33^ The CCK is an essential attribute for the structure of cyclotides, but little is known about its role on bioactivity.^22,34,35^ The current study explores the functional role of the CCK motif using the kalata B1 analog, and clinical drug candidate T20K, as a prototypical cyclotide. Several partly reduced or wrongly formed cystine knot variants were prepared by chemical synthesis and were assayed for antiproliferative activity on peripheral blood mononuclear cells (PBMCs) relative to the native folded peptide. Furthermore, the contribution of the cyclic backbone was characterized using a linearized version of the cyclotide, leading to the finding that both an intact cystine knot and a cyclic backbone are vital structural elements for the biological activity of T20K as an immunosuppressant peptide. These observations were explained at a structural level by using NMR spectroscopy and at a cellular level by determining the cellular uptake of native vs linearized T20K.

## Materials and Methods

### Chemicals

Formic acid (LC–MS grade), ammonium hydrogen carbonate, bovine trypsin (sequencing grade), dithiothreitol (DTT) (biochemistry grade), citrate dihydrate salt (biochemical grade), and calcium dichloride were from Sigma Aldrich (St. Louis, United States). Dichloromethane (DCM), acetonitrile (AcN) (HPLC and LC–MS grade), methanol (MeOH) (HPLC synthesis or gradient grade), water (LC–MS grade), and 2-propanol (2-PrOH) (LC–MS grade) were from ChemLab (Bensheim, Germany). Trifluoroacetic acid (TFA), Tris-(hydroxymethyl)-aminomethane (Tris), glycine, sodium chloride (NaCl), acetic acid, and reduced or oxidized glutathione (GSH or GSSG, respectively) were from Carl Roth (Karlsruhe, Germany). Endoprotease ArgC was from Abnova (München, Germany).

### Chromatographic Separation

Peptide separation was achieved on a Dionex Ultimate 3000 HPLC system (Thermo Scientific) in the reversed phase chromatography mode. Chromatographic columns used were a Dichrom Kromasil C_18_ column (250 × 20.2 mm, 10 μm), Dichrom Kromasil C_18_ column (250 × 10 mm, 5 μm), Dichrom Kromasil C_18_ column (250 × 4.2 mm, 5 μm), or Phenomenex Kinetex C_18_ column (150 × 3 mm, 2.1 μm) at 8, 4, 2, and 0.3 mL min^–1^, respectively. The mobile phase was 0.1% (v/v) trifluoroacetic acid (TFA) in ddH_2_O for eluent A and acetonitrile/ddH_2_O/TFA 90/10/0.09% (v/v/v) for eluent B. If not otherwise stated, chromatographic separations used the following gradient: a hold of 5% B for 5 min, a gradient from 5 to 65% B (steepness 0.5, 1.0, 2, or 3% eluent B min^–1^), followed by a wash and an equilibration phase. Peptide elution was monitored with UV detector traces at 214, 254, and 280 nm. For fraction collection, an automatic fraction collector (ThermoFisher) was used with fixed time increments for collection. All peptide fractions were lyophilized and analyzed via MALDI-TOF mass spectrometry (MS).

### Mass Spectrometry

MALDI-TOF analysis of peptides was performed with an Autoflex MALDI-TOF MS analyzer (Bruker Daltonics). All spectra were prepared in the positive reflector mode. The mass spectrometer was daily calibrated with five-point calibration using a quadratic function in the internal calibration mode with Peptide Mix 4 from LaserBiolabs (Sophia-Antipolis, France). The matrix α-cyano-hydroxy-cinnamic acid, dissolved in acetonitrile/ddH_2_O/TFA 50/50/0.1% (v/v/v), was mixed with the sample in a ratio of 6:1. A 0.5 μL aliquot of the mixture was spotted on the MTP 384 ground steel target plate. LC–MS experiments applied a chromatographic separation on an Ultimate 3000 RSLC HPLC using a PepMap Acclaim RSLC column (250 mm × 75 μm, 2 μm) (both from Thermo Scientific). The two-dimensional system was equipped with a pre-column for pre-concentration and desalting using 0.1% TFA as a mobile phase. The sample separation was achieved with 4 μL min^–1^ with eluent A 0.1% aqueous formic acid and eluent C acetonitrile/ddH_2_O/formic acid 80/20/0.08% (v/v/v). Linear elution gradients from 5 to 65% eluent C, with an increment of 0.5, 1.0, or 2% eluent C/min were applied for peptide separation with a flow rate of 4 μL/min. The chromatography system was coupled to a QqTOF mass spectrometer oTOF compact from Bruker Daltonics (Billerica, MA, United States) using the microflow ESI source in the positive ionization mode. Data analysis used oTOF control software v3.4 (build16). The device was externally calibrated in the enhanced quadratic mode using the low concentration calibration mix (Agilent Technologies) in the range of 118 to 2200 Da before starting sequential analysis. Additionally, high mass accuracy was achieved by an internal calibration based on the lock mass calibration with the calibrant hexakis-(1*H*,-1*H*,-4*H*-hexafluorobutyloxy)-phosphazine (Agilent Technologies). The source and ion transfer parameter were optimized over the whole mass signal range to give maximal signal, i.e., 4500 V capillary voltage, 0.5 L min^–1^ nitrogen nebulizer flow, and 5 L min^–1^ dry gas glow at 180 °C. All mass spectra were recorded in the positive ionization mode monitoring TIC traces. The peptide compound specific [M + *n*H]^*n*+^ mass signals used for analysis or quantitation are summarized in Table 1.

**Table 1:** Chemical and Mass Spectrometric Parameters of T20K Peptides

**name**	**specification**	**molecular formula**	**molecular weight** [g/mol](monoisotopic)	**[M + H]**^+^(theoretical)	**[M + 3H]3+**
T20K	cyclic native oxidized	C119H184N36O38S6	2917.19	2918.20	973.40
cyclic reduced T20K	cyclic cysteine reduced	C119H190N36O38S6	2923.24	2924.24	975.42
T20K-Acm	-*S*-acetamide	C131H208N42O44S6	3265.36	3266.37	1089.46
T20K-S-methyl	-*S*-*S*-methyl	C125H202N36O38S12	3199.16	3200.17	1067.39
T20K-Nem	-*S*-(*N*-ethyl-maleimide)	C155H232N42O50S6	3673.52	3674.53	1225.51
1SS variant	cystine knot truncated; four *S*-acetamides	C127H200N40O42S6	3149.31	3150.31	1050.78
2SS variant	cystine knot truncated; two *S*-acetamides	C123H192N38O40S6	3033.25	3034.26	1012.09
T20K-(Cys to Ala)	all cysteines changed to alanine	C119H192N36O39	2731.40	2732.41	911.47
3SS variants of T20K	cyclic non-native oxidized cystine knot	C119H184N36O38S6	2917.19	2918.20	973.40
linear folded T20K variant	linear (Arg|Asn) native folded	C119H186N36O39S6	2935.20	2936.21	979.41
T20K-CF	Lys20-ε-amine labeled carboxyfluoresceine	C140H192N36O44S6	3275.63	3276.64	1092.89

### Peptide Synthesis and Oxidative Folding

Fmoc solid-phase peptide synthesis of cyclic or linear T20K used established synthesis strategies similar as described in a previous work.^22,36^ Cleavage of the peptides from the resin and side-chain deprotection were carried out in a mixture of TFA:triisopropylsilane (TIPS):H_2_O 95:2.5:2.5, (v/v/v) for 2.5 h at room temperature. The peptides were purified by RP-HPLC on the preparative system with a linear gradient from 5 to 65% eluent B over 60 min and a flow rate of 8 mL min^–1^. Oxidation and folding were performed with standard conditions if not stated differently elsewhere: ∼0.5 mg/mL peptide in 0.1 M NH_4_HCO_3_ buffer solution (pH 8.3) containing 50% 2-PrOH, 2 mM GSH and 0.5 mM GSSG for 24 h at room temperature (standard conditions for oxidative folding). The reaction was stopped by acidification, and the folded peptides were isolated by HPLC separation. Native folded peptide T20K has increased retention on reversed phase columns compared to fully reduced peptide or folding intermediates. The peptide peak shift allowed clear separation of HPLC fractions. The folding products were purified using HPLC, and their identity was confirmed by retention time comparison with a reference material or by mass spectrometry comparing the detected mass signal with the theoretical *m*/*z* value. For the preparation of non-native folded peptides, nine different folding conditions were analyzed (summarized in Table S1). The optimized condition (0.1 M NH_4_HCO_3_, pH 8.5 containing 50% DMSO) was applied for the chemical preparation of non-native 3SS variants of T20K. The variants were purified by HPLC using linear 120 min gradients. The 3SS variants had baseline or partial overlapping peak elution. A retention time index (RI) was applied to distinguish these variants according to eq 1. The RI was calculated based on the retention time of three reference points, i.e., the fully reduced peptide, the native folded T20K, and the 3SS variant of interest using linear gradients in reversed phase separations. The concentrations of peptide solutions were calculated using the Beer–Lambert law at *A*_280_ (ε_[T20K]kB1_ = 6410 L·M^–1^·cm^–1^).
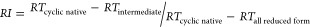
1

### Peptide Modifications

Cyclic folded peptide was dissolved in reaction buffer 0.1 M NH_4_HCO_3_ (pH 7.8).

For the preparation of 1SS and 2SS T20K variants, the peptide solutions were treated with dithiothreitol (DTT) in submolar concentrations (0.25-fold) based on the calculated molar content of cysteines. Disulfide bond reduction was carried out at 37 °C for 1 h, and the conversion product was evaluated with mass spectrometry. For alkylation of free sulfhydryl groups, the reduced peptide was treated in a 10-fold molar excess of the reactive reagent vs reducing agent for 10 min in the dark. The alkylant was quenched with additional DTT and diluted with HPLC running buffer. HPLC fractionation with 30 s/sample using conditions described in the corresponding section was applied for the preparation of HPLC fractions with 1SS and 2SS peaks. The peptide concentration of the resulting fractions was determined on a nanodrop spectrophotometer. A 50 μM solution of each sample was evaluated with mass spectrometry and analytical HPLC. 1SS and 2SS folding variants were isolated with an analytical Kromasil column using a separation gradient of 5 to 65% eluent B in 0.5% min^–1^. Analyte peaks were manually collected, and the purity of peptides was evaluated by mass spectrometry and HPLC. Fully reduced T20K was obtained with 10-fold molar excess of the reducing agent under the previously described conditions. Purification was performed with C_18_-modified solid phase extraction (SPE) cartridges (Phenomenex). The peptides were lyophilized and stored until further use at −20 °C. To avoid refolding of the reduced peptides, the analyte was solubilized for any subsequent steps in an acidified solution of 0.01% acetic acid (pH < 5). The derivatization with iodoacetamide, with methyl methanethiosulfonate (MMTS, dissolved in EtOH; Sigma Aldrich) and with *N*-ethylmaleimide (Nem, dissolved in EtOH; ThermoFisher) was performed with 10-fold reagent excess to reactive sites in the peptides (e.g., 6 cysteine = 6 × 10-fold the molar peptide concentration) at room temperature for 10 min in the dark or for Nem and MMTS within 2 h, respectively. The reaction was stopped with the addition of 50% TFA solution to a final pH of 2–3. For the preparation of the linear native folded peptide, the SPE-purified cyclic fully reduced variant was subjected to site-specific proteolytic cleavage. Clostripain (endoproteinase ArgC) was applied in 0.05 M NH_4_HCO_3_ pH 7.8 with 1 mM CaCl_2_ and 1 mM DTT at 22 °C for 2–4 h. The peptide:protease ratio was 1:50, and the conversion of cyclic-reduced to the linear-reduced peptide (Δ + 18 Da for hydrolyzation) was monitored with mass spectrometry. A total of 10 mM DTT was added to the reaction for 10 min at 60 °C to account for a cysteine oxidation during the cleavage reaction. The sample was acidified to stop the reaction, and the buffer was exchanged with a C_18_ SPE clean-up. Subsequently, the oxidative folding in standard folding buffer was initiated for 24 h. The linear peptide T20K (opened in loop 6) folded properly within a short time providing a peak with retention time shift similar to that observed for native folded peptides. For structural analysis, the linear T20K variant was also prepared by peptide synthesis and the folded peptide was identical by retention time as analyzed by HPLC to the linear T20K prepared by enzymatic cleavage (Figure S7C).

### Characterization of Cystine-Knot Variants of T20K

For the mapping of cystine connectivities, the 1SS and 2SS variants were reduced with 5 mM TCEP in 0.1 M citrate buffer pH 3.6 at 37 °C for 1 h. The sulfhydryls were derivatized with 25 mM Nem at 25 °C for 10 min, and the reaction was stopped with 5 mM glutathione. The sample pH was adjusted to ∼7.8 with 0.2 M NH_4_HCO_3_ buffer and tryptic digestion was performed at 37 °C for 4 to 24 h. The tryptic fragments were analyzed with mass spectrometry, recording [M + H]^+^ signals at the MS1 stage. Tryptic peptides were characterized by *de novo* amino acid analysis of MS/MS fragmentation spectra. The cystine connectivity assignment of 1SS and 2SS variants was rationally derived from the determined *S*-acetamides and *N*-ethyl-maleimide alkylation cysteine residues (summarized in Table S2). To assign the cystine connectivity of 3SS variants of T20K, the compounds were partially reduced and alkylated in two consecutive steps and the alkylation patterns of two species determined by mass spectrometry. The peptides were diluted to 100 μM (50 μL) in 0.2 M citrate buffer pH 3.6. The reduction was carried out with 0.2–2 mM TCEP at 55 °C for 3 min. The samples were diluted with eluent A and immediately injected for HPLC separation. An analytical Kinetex column was used with a 1 or 3% min^–1^ eluent B gradient similar to that described above. Collected peaks were analyzed by MALDI-MS to identify 1SS (*m*/*z* 2922.23) and 2SS (*m*/*z* 2920.21) variants. The lyophilized peptides were solubilized in 10 μL of 10 mM Nem in citrate buffer, and the reaction was allowed to proceed for 10 min in the dark. Sample alkylation was evaluated with mass spectrometry by monitoring the mass signals for 1SS (*m*/*z* 3422.79) and 2SS (*m*/*z* 3170.49) species. The sample was diluted with 30 μL of 5 mM DTT in 0.3 M NH_4_HCO_3_ buffer with pH 7.8, and the samples were incubated at 55 °C. The mixed alkylated species of the 3SS5 variant were purified by HPLC to ensure unambiguous disulfide assignment. The full reduction was monitored by mass spectrometry, and after approximately 20 min, the partially Nem-alkylated and fully reduced analytes were obtained and the alkylation with 25 mM IAM was carried out at room temperature for 10 min. The reaction was quenched with 5 mM DTT, and mass spectrometric analysis confirmed mixed alkylated 1SS (*m*/*z* 3538.83) and 2SS (*m*/*z* 3402.61) peptides. A tryptic digestion was performed obtaining cleavage fragments (NGLPVCGETCVGGTCNTPGCK and CSWPVCTR) with mixed cysteine alkylation patterns, which were characterized by *de novo* amino acid analysis using MS/MS fragmentation data. The assigned disulfide connectivities are summarized in Table S3. The analyzed *m*/*z* mass signals and the identified alkylation pattern of tryptic fragments, which were used for the cystine connectivity assignment of 3SS variants of T20K are depicted in Table S4.

### Isolation and Cultivation of Human Peripheral Blood Mononuclear Cells (PBMC)

Blood of healthy donors was obtained from the Blood Transfusion Centre (University Medical Centre Freiburg, Freiburg Germany), and all experiments conducted on human material were approved by the ethics committee of the University Freiburg (55/14). Venous blood was centrifuged to isolate peripheral blood mononuclear cells on a LymphoPrep gradient (density: 1.077 g/cm^3^, 20 min, 500 × *g*, 20 °C; Progen, Heidelberg, Germany). PBMCs were collected and washed twice with phosphate buffer saline (PBS; GE Healthcare, München), and cell viability and concentration were determined using the trypan blue exclusion test. Cells were cultured in RPMI 1640 medium supplemented with 10% heat-inactivated fetal calf serum, 2 mM l-glutamine, 100 U/mL penicillin, and 100 U/mL streptomycin (all from Life Technologies, Paisley, UK) and cultured at 37 °C in a humidified incubator with a 5% CO_2_/95% air atmosphere.

### Analysis of Lymphocyte Proliferation

Harvested PBMC were washed twice in cold PBS and resuspended in PBS at a concentration of 5 × 10^6^ cells per mL. Carboxyfluorescein diacetate succinimidyl ester (CFSE; 5 μM; Sigma-Aldrich, St. Louis, MO) staining was performed to determine T-cell proliferation. Cells were stained with CFSE and incubated for 10 min at 37 °C. The staining reaction was stopped by washing twice with complete growth medium. Afterward, stained cells were stimulated with anti-human CD3 (clone OKT3) and anti-human CD28 (clone 28.6) mAbs (each 100 ng/mL; both from eBioscience, Frankfurt, Germany) treated as indicated in the figure legends, with medium alone, cyclosporine A (CsA; 5 μg/mL, purity ≥99%, Sandimmun 50 mg/mL, Novartis Pharma, Basel, Switzerland), camptothecin (CPT; 300 μM; purity >98%; Tocris, Bristol, UK), or in the presence of the indicated concentrations of peptides for 72 h at 37 °C in a humidified incubator with a 5% CO_2_/95% air atmosphere. Cell division progress was analyzed by flow cytometric analysis using a FACS Calibur analyzer (BD Bioscience, Becton Dickinson, Franklin Lakes, NJ). Data were generated using FlowJo software. For further analysis of FACS raw data Microsoft Excel and SPSS software (IBM, Version 22.0, Armonk, USA) were applied. All bioactivity data are represented as mean ± standard deviation (SD) for the indicated number of independent experiments. Statistical significance was determined by a one-way ANOVA followed by Dunnett’s post hoc pairwise comparisons. The asterisks (*p < 0.05, **p < 0.01, ***p < 0.001) represent significant differences from the respective control.

### Label-Free Quantification of Peptide Uptake into Jurkat Cells

The uptake of peptides T20K, 3SSV5, and linear (Arg|Asn)T20K was studied with label-free quantitation using mass spectrometry. The samples were analyzed with an Acclaim PepMap RSLC C_18_ 150 × 0.3 mm 2 μm 100 Å as described in the previous section or with a Phenomenex Kinetex C_18_ 150 × 2.1 2.6 μm, 110 Å column utilizing the 280 nm UV trace for detection. Post-acquisition data analysis was performed in DataAnalysis software v4.2 and the QuantAnalysis (from Bruker Daltonics). An external calibration with 43.76, 21.88, 10.94, 5.47, 2.74, 1.37, 0.68, 0.34, 0.17, and 0.085 μg/mL was used together with the mass trace *m*/*z* 973.40 for the quantitation of all T20K variants. A quadratic calibration function with a 1/*X* weighing provided the best fit for the entire calibration range applying the LC–MS system, whereas for the HPLC-UV measurement, the linear calibration function was for a reduced calibration range. A total of 2.0 × 10^6^ Jurkat cells per experiment were incubated with 10 μM (∼29.17 μg/mL) peptide. A time course study was conducted with 0, 10, 20, 40, 60, and 120 min incubations, measuring the concentration of the peptide in whole cell extracts and in serum-free RPMI medium (supernatant). All experiments were carried out in triplicate replications, and data are represented as mean with standard deviation. A PBS control and a recovery sample (serum free RPMI medium) were included to account for the unspecific loss of peptide with a determined recovery. The cells were pelleted from the RPMI growth medium by centrifugation with 400 *g* for 5 min. The cells were washed two times with 100 μL PBS, and the washing fraction including the medium will be referred to as the cell supernatant sample. Whole cell extracts were prepared similar to that described previously in Henriques and Craik^37^ using 500 μL acetonitrile and a cell membrane disruption with a 2 min ultrasonic treatment. Insoluble cell material was separated from the clear supernatant by centrifugation with 16,000 *g* at 4 °C for 20 min. The acetonitrile extract was evaporated under vacuum, and the sample was solubilized in 100 μL buffer acetonitrile/water/formic acid 10/89/1% (v/v/v). The soluble part was transferred into silanized microinjection vials, and 7 or 20 μL was injected to the MS or the UV system, respectively. The determined analyte concentration of the measurement sample is illustrated as the amount of peptide per 200,000 cells derived from the whole cell extract.

### Fluorescence Microscopy

T20K was labeled with *N*-hydroxysuccinimide-5/6-carboxy-fluorescein (Santa Cruz Biotechnology). The peptide was dissolved in 0.1 M NaHCO_3_/Na_2_CO_3_ pH 8.6 and incubated for 8 h at 22 °C with a 20-fold molar excess of the labeling reagent (prepared in anhydrous dimethylsulfoxide; Sigma Aldrich). The reaction was quenched with 0.1% (v/v) TFA, and the labeled peptide product (T20K-CF) was isolated via RP-HPLC and lyophilized. Following quality control via MS, the labeled peptide was dissolved in PBS, and its concentration determined by nanodrop measurement at 490 nm (fluorescein absorption maxima, ε_490_ = 75,000 mol^–1^ × cm^–1^). Internalization of the carboxy-fluoresceine peptide T20K-CF into Jurkat cells was analyzed in a concentration- and time-dependent manner using 1, 4, and 10 μM peptide, and 1 or 24 h of incubation. Afterward, cells were additionally stained with CellMask Deep Red (0.5 μg/mL; Life Technologies, CA, United States), propidium iodide (1 μg/mL, Sigma Aldrich), or Hoechst 33342 dye (10 μg/mL; from Sigma Aldrich) to visualize the cell membrane and nucleus, respectively. Cells were imaged with a Zeiss LSM 510 (Carl Zeiss Microscopy GmbH, Jena, Germany) and with a Nikon Eclipse Ti A1 inverted microscope system, respectively. The main beam splitter was set to HFT UV/488/543/633 nm. An argon/2 laser 488 nm and a HeNe 594 nm were used for excitation. Excitation (Ex) and emission (Em) settings were as follows: fluorescein Ex 488 nm/Em 500–530 nm; CellMask Orange Ex 554 nm/Em 565–615 nm band pass filter; or CellMask Deep Red Ex 633 nm/Em 650 (long pass filter). Prior to imaging, cells were washed with PBS to remove excess of dye, and the cells were incubated in phenol red-free growth medium. Scale bars of cropped images were manually created, processed, and added with the Fiji processing software.^38^

### NMR Spectroscopy and Structural Analysis

T20K and linear T20K (both obtained by peptide synthesis) were dissolved in H_2_O/D_2_O (10:1, v/v) at a concentration of 1 mM, and pH 3.3. NMR spectra were acquired on a Bruker Avance III 600 MHz NMR spectrometer, including 1D ^1^H spectra and 2D TOCSY, NOESY, and ^1^H-^15^N HSQC measured at 298 K. Solvent suppression was achieved using excitation sculpting, and 2,2-dimethyl-2-silapentone-5-sulfonate (DSS) was used as an internal standard at 0 ppm. To enable full structural characterization of both peptides, additional TOCSY spectra were acquired at 283–308 K to measure the sensitivity of amide shifts to temperature. Peptides were also dissolved in 100% D_2_O for deuterium exchange experiments and acquisition of ECOSY and ^1^H-^13^C HSQC spectra. Spectra were processed using Topspin 3.5 (Bruker) and assigned with CcpNmr Analysis.^39^ A total of 278 and 198 distance restraints, derived from cross peaks in NOESY spectra (mixing time of 200 ms), were used to generate preliminary structures of T20K and linear T20K, respectively, with CYANA 3.97, along with disulfide bond restraints between Cys I-IV, II-V, and III-VI. Based on the assigned chemical shifts, TALOS-N predictions of torsion angle restraints (18 phi and 14 psi for T20K; 17 phi and 7 psi for linear T20K) were added to the calculations.^40^ Seven and three chi1 side-chain angle restraints were also included as predicted by ECOSY and NOESY data for T20K and linear T20K, respectively. Analysis of preliminary structures, amide temperature coefficients, and deuterium exchange experiments allowed the addition of 14 restraints for 7 hydrogen bonds in T20K, and 6 restraints for 3 hydrogen bonds in linear T20K. Final sets of structures were then generated in CNS using torsion angle dynamics, refinement, and energy minimization in explicit solvent.^41^ Stereochemical quality of final structures were assessed using MolProbity.^42^ The structural coordinates were submitted in the wwPDB (PDB ID 7LHC, T20K; PDB ID 7RFA, linear T20K), and chemical shifts were deposited in the Biological Magnetic Resonance Bank (BMRB ID 30848, T20K; BMRB ID 30935, linear T20K).

### Sequence Logo Preparation

A sequence logo was prepared using the webtool Weblogo 2.82 and published sequences of immunosuppressive cyclotides: kB1 and amino acid mutants of it (T20K, G18K, N29K, T20K, N29K), pase peptides A–E, cycloviolacin O1, -O3, -O28, -O32, -T, kalata B2, -B5, -S, vaby B, vibi 6, -E, -K and varv peptide C.^11,21,43^ The sequence plot shows frequencies of residues at position 1-33 of the core region from cyclotide precursor genes.

## Results

### Contribution of the Cystine Knot to the Immunosuppressive Activity of Cyclotides

As a representative cystine knot peptide, we chose to study the cyclotide T20K due to its importance as a clinical drug candidate for multiple sclerosis.^19,20^ After assembly by solid phase peptide synthesis, the native cystine knot was obtained by oxidative folding, which was monitored by RP-HPLC/MS. A recent study demonstrates that certain residues and surface properties are crucial for the biological activity of T20K, whereas other residues are amenable to chemical modification.^21^ Aside from this initial mutational study, the role of the CCK motif for immunosuppressive activity in not known (Figure 1A,B).^44^ Hence, we utilized the immunosuppressive activity of T20K as a model to decipher the functional importance of its unique CCK motif.^18,19,21^ T20K exhibits a concentration-dependent antiproliferative effect on T-cells with a half maximal inhibitory concentration (IC_50_) of ∼6 μg/mL (∼2 μM).^21^ This concentration has a comparable effect to the reference compound cyclosporine (CsA) at 5 μg/mL.

**Figure 2:**
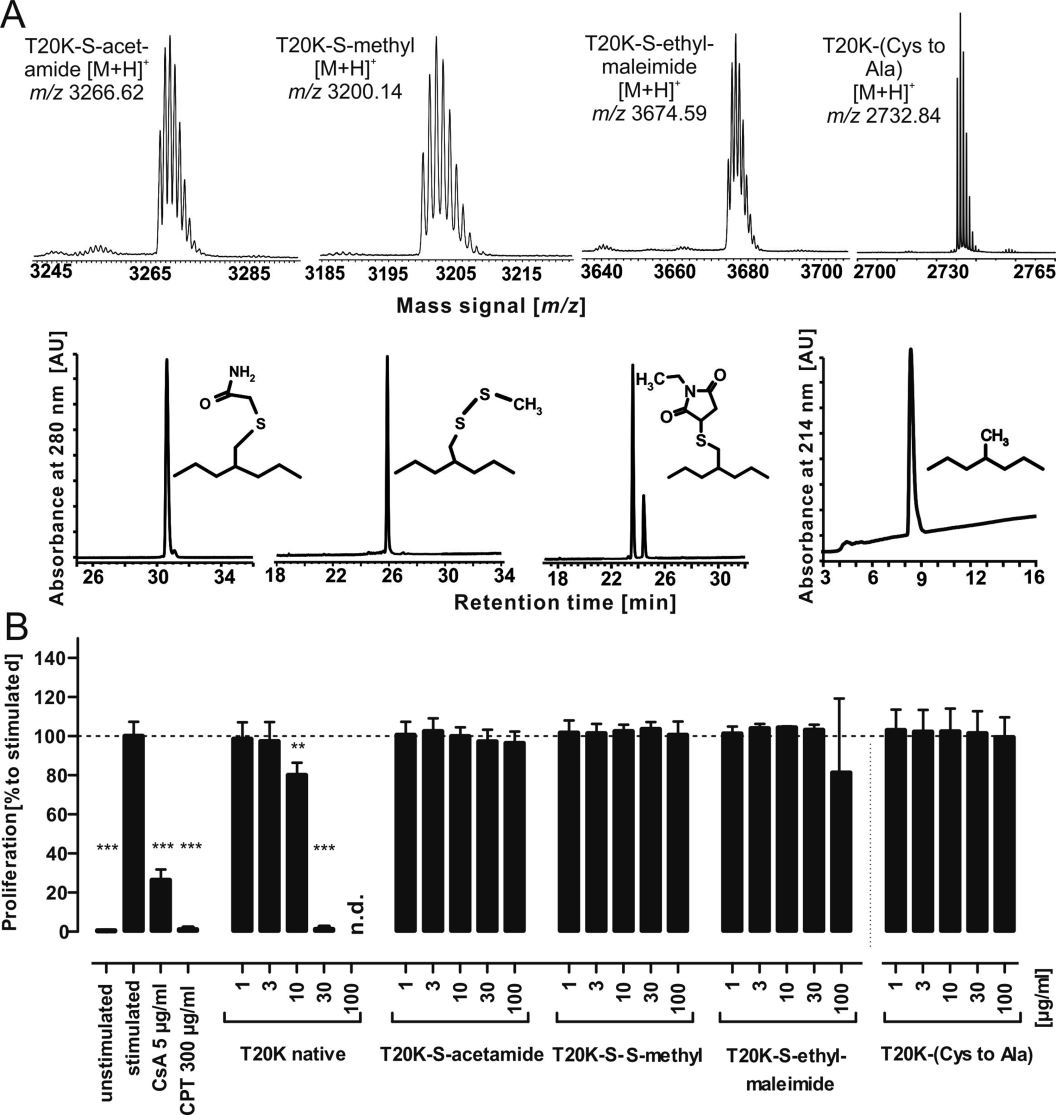
Cystine knot truncated variants. (A) T20K was reduced with dithiothreitol, and free-reactive sulfhydryl moieties were derivatized with site-specific reagents iodoacetamide, methyl methanethiosulfonate, and *N*-ethyl-maleimide, providing the *S*-acetamide, the -*S*-*S*-methyl, and the *S*-ethyl-maleimide derivative, respectively. Following HPLC purification, the identities of any derivative were confirmed by mass spectrometry. Additionally, the probe T20K-(Cys-to-Ala), where all six cysteines were replaced with the isostere alanine, was prepared to rule out steric hindrance of sulfhydryl derivatization. (B) All three cysteine-derivatized peptides as well as the variant with isosteres showed a total loss of antiproliferative activity in the tested concentration range of 1–100 μg/mL peptide. All data represent the mean ± standard deviation of three biological replicates, expressed relative to the stimulated control (≙100%), The data for the T20K-(Cys-to-Ala) were normalized to the stimulated control shown in Figure S1. CsA and CPT are positive controls. Asterisks (**p < 0.01, ***p < 0.001) indicate significant differences compared to stimulated control, and (n.d.) indicates data not detected.

A cystine knot-deficient T20K peptide, denoted as the reduced variant, was prepared by the chemical reduction of the cysteine residues (Figure 1C). Upon unfolding of the knot, the peptide lost its antiproliferative activity toward human PBMCs (Figure 1B). Reduced peptides carry unconjugated sulfhydryl groups, and these moieties are prone to conjugation during the assay. Hence, T20K was derivatized with thiol-reactive reagents to obtain *S*-acetamides (using iodoacetamide, -Acm), -*S-S*-methyls (using methyl methanethiosulfonate, -*S*-methyl) as well as the *S-*(*N*-ethyl-maleimides) using *N*-ethyl-maleimide (Nem) (Figure 2A). A total of ∼30 μg/mL native T20K is sufficient to completely inhibit immune cell proliferation. In contrast, the three sulfhydryl modified peptides did not affect cell proliferation in the tested concentration range of 1–100 μg/mL (Figure 2B). A control peptide with all cysteines replaced by alanine exhibited no activity in the bioassay, as expected. This implied that the native cystine knot is an integral functional part of the peptide. Consequently, it was important to find out if all three disulfide bonds are equally important for bioactivity and how proliferation of T-cells is affected by modifications of the CCK.

### Biological Characterization of Partial Unfolded Cystine Knot Variants

The hypothesis was that a partially unfolded cystine knot, in which one or two cysteines were eliminated by partial reduction and alkylation, would partly be able to rescue immunosuppressive activity. Therefore, T20K variants, denoted as 1SS and 2SS, were prepared by reducing one or two disulfide bonds. The reduced sulfhydryl groups were derivatized as stable *S*-acetamide moieties to avoid thiol-reshuffling or oxidative refolding. Hence, the remaining disulfides of these variants should remain in the native configuration.^45^ Up to eight samples with mixtures of partially unfolded T20K containing either 1SS (*m*/*z* 3150.31) and/or 2SS (*m*/*z* 3054.26) variants were identified and semi-purified by RP-HPLC and MS (Table 1, Figure S1A–C). Six of the identified probes, three 1SS and three 2SS variants, were isolated to purity to allow chemical characterization and bioactivity evaluation (Table S2). To confirm the cystine connectivity of the isolated 1SS and 2SS variants, we evaluated the *S*-acetamides and *S-*(*N*-ethyl)-maleimide-mixed alkylated partial unfolded peptides via tryptic cleavage and MS/MS. The *de novo* assignment of peptide fragmentation ion signals enabled the identification of the C_I-IV_, C_II-V_, and C_III-VI_ (1SS) as well as the C_II-V_/C_III-VI_, C_I-IV_/C_III-VI_, and C_I-IV_/C_II-V_ (2SS) variants (Figures S2 and S3; Table S2). Results from the bioassay indicated that the probes with a partially truncated cystine knot topology were inactive over the entire tested concentration range (Figure S1D), suggesting that T20K analogues bearing any chemical modification to the cystine knot (e.g., *S*-acetamide, *S*-methyl, maleimides, or cysteine-to-alanine replacement) or partial unfolding of the cysteine knot have lost the ability to elicit any anti-proliferative activity on PBMCs.

**Figure 3:**
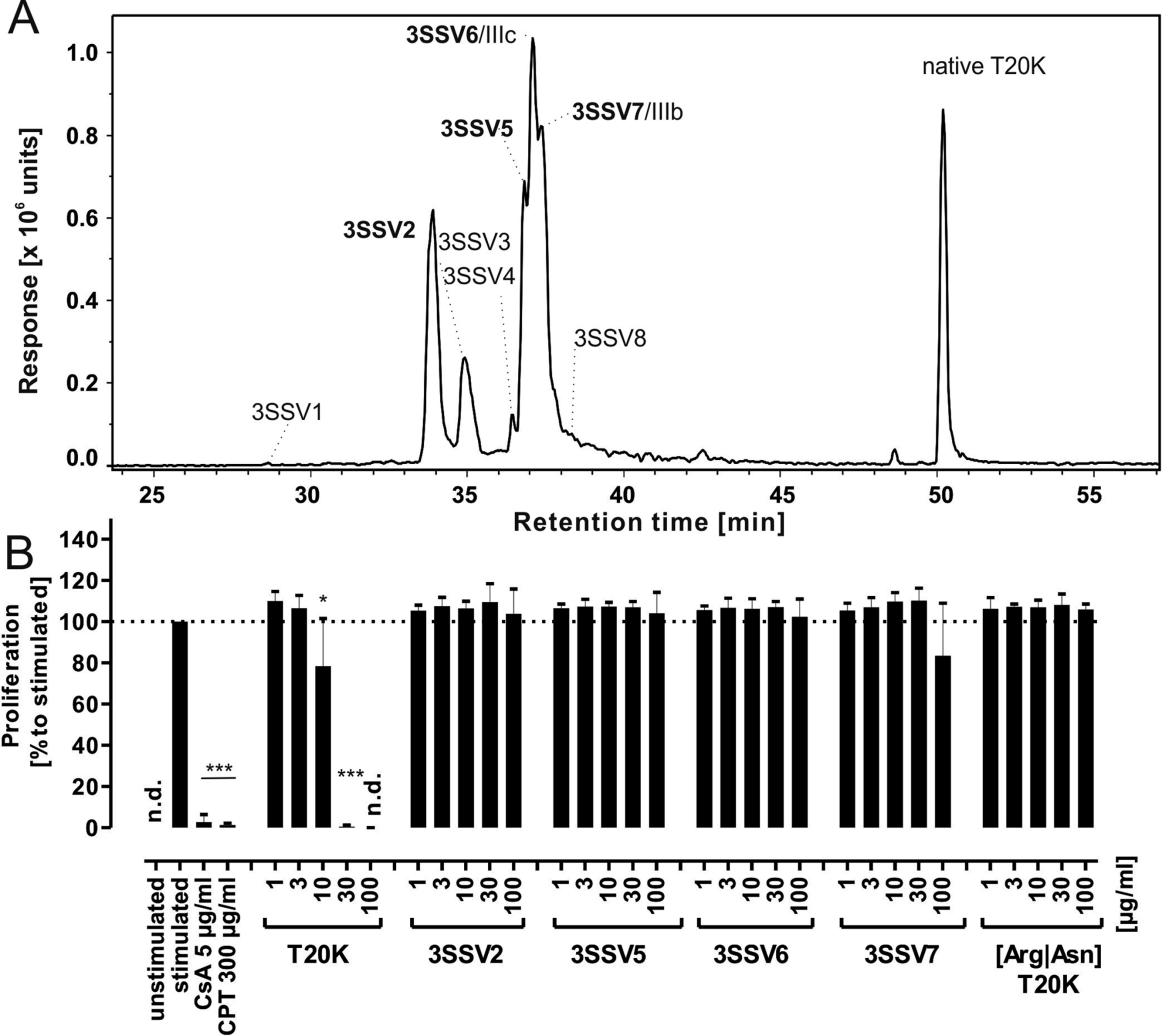
Non-native folded 3SS variants and linear-folded variant. (A) LC–MS analysis of the EIC mass trace *m*/*z* 973.40 (width ± 0.02) for the [M + 3H]^3+^ signal of T20K derived 3SS variants. The spectral data were smoothed using a Savitzky Golay function (smoothing width 4.2 s and two data points). Fully reduced peptide was incubated in folding buffer 6 for up to 96 h. The non-native 3SS variants were generated in folding buffer, along with native folded T20K. Peaks were labeled in order of elution. Bold type denotes those species tested in the antiproliferative assays. (B) All tested 3SS variants showed abolished bioactivity over the entire tested concentration range compared to T20K. The linear-folded (Arg|Asn)T20K variant had no antiproliferative activity up to a concentration of 100 μg/mL. The data set represent mean ± standard deviation of three biological replicates, expressed relative to stimulated control (≙100%). Asterisks (*p < 0.05, ***p < 0.001) indicate significant differences compared to stimulated control, and (n.d.) indicates data not detected.

With these data in mind, we tested whether fully oxidized but misfolded variants of T20K, denoted as 3SS, would be immunosuppressive, and so we prepared T20K variants containing a non-native cystine knot. The formation of these 3SS variants was empirically determined by testing several conditions for oxidative folding of T20K (Table S1). As the expected isoforms were isobaric with a corresponding mass signal of *m*/*z* 2918.14, the 3SS variants were analyzed via RP-HPLC (Figure S4). We assigned retention indices (RI) for each variant to uniquely assign these peptides (eq 1) and compare them to earlier studies. In theory, there are 15 possibilities to form three disulfide bonds in a cystine knot peptide. During the oxidative folding process, eight 3SS peptides were identified by LC–MS along with transient 1SS- and 2SS folding intermediates or dead-end folding side products, similar to that in previous studies.^7,46^ Four of the 3SS variants were purified in sufficient quantities (Figure 3A; Figure S5, Table S3), and their cystine connectivity was determined as described for the partial unfolded variants. Mixed alkylated probes were generated by partial reduction of the non-native folded cystine knot and a twostep alkylation of 1SS or 2SS variants. The alkylation pattern of these species was determined by MS/MS fragmentation and *de novo* amino acid assignment using tryptic peptides (Figure S6, Table S4). Concentration-response experiments of these 3SS variants exhibited no effect on the proliferation of activated PBMCs up to a concentration of 100 μg/mL (Figure 3B). These cysteine truncation experiments indicated that any modification of the native cystine knot is detrimental to the immunosuppressive activity of T20K and highlighted the unique role of the native cystine knot configuration of cyclotides.

### Role of the Cyclic Backbone in Antiproliferative Activity

After dissecting the role of the cystine knot, we evaluated the contribution of the cyclic backbone as a component of the CCK motif. Acyclic but native folded T20K variants were prepared. Cyclic reduced T20K was incubated with endoproteinase ArgC to yield a linear cleavage product via site-specific proteolytic activity at the Asn residue in loop 6 (GLPVCGETCVGGTCNTPGCKCSWPVCTR↓N, where ↓ denotes the cleavage site). The linearized peptide derived from ArgC proteolysis was prepared, and oxidative folding yielded a late eluting peak with an increased retention time compared to folding intermediates and other 3SS species and an *m*/*z* of 2936.21 corresponding to the linear folded peptide (Figure S7A). Next, the linear [Arg|Asn]T20K peptide was assayed in T-cell proliferation experiments (Figure 3B). Similar to the reduced or non-native cystine knot variants, the linearized native folded T20K variant had no antiproliferative effect on PBMCs up to a concentration of 100 μg/mL, suggesting that not only the cystine knot but also the intact cyclic backbone is essential for activity of T20K. Knowing that only the native cyclotide is immunosuppressive, we investigated possible consequences of backbone linearization or cystine knot unfolding of T20K for the interaction with and the localization in immune cells.

### Cellular Uptake and Localization of T20K in Immune Cells

**Figure 4:**
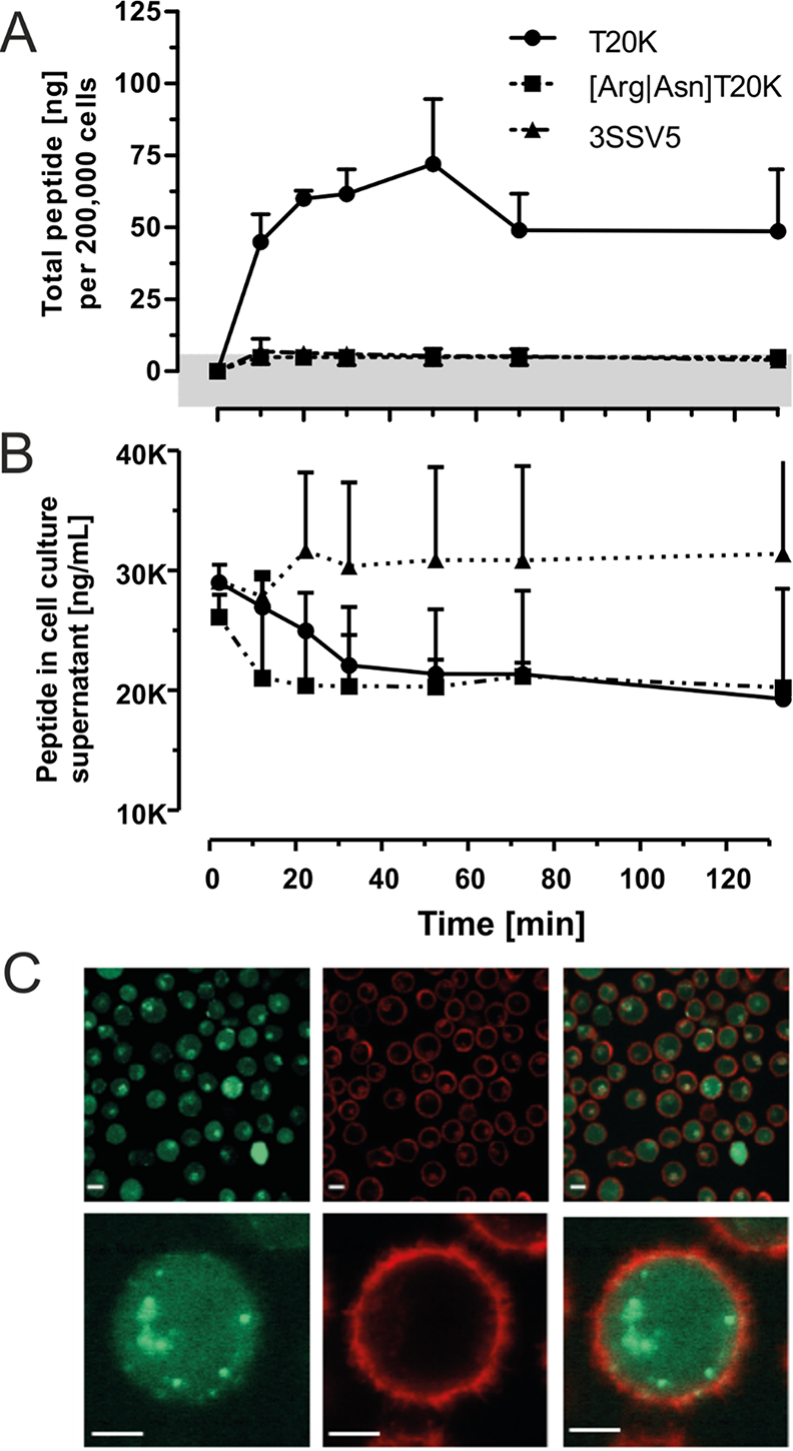
Cellular uptake of T20K into mammalian immune cells. Jurkat cells were incubated with native T20K as well as with 3SSV5 and linear T20K variants in a concentration of 10 μM (e.g., 21.17 μg/mL for the native species) in serum-free RPMI medium for various time points. The cell supernatant and cell pellet were harvested, and the peptides were extracted with acetonitrile. The lyophilized extracts were analyzed via LC–MS or HPLC-UV. (A) Whole cell extracts were analyzed with LC–MS to quantify analytes in absolute concentrations. The peptide levels determined for native T20K were in the nanogram range, whereas non-native peptide variants were not detected above the determined quantitation limit of 85 ng/mL (indicated by a grey horizontal bar). All data are provided as the mean of triplicates with standard deviation, except of the linear variant, which was analyzed two times. (B) The level of native T20K in cell supernatants revealed a decreasing trend overtime, which may account for the accumulation of the peptide in the cells. The levels of the other probes were not altered; both, the linear and the 3SSV5 variant remained at a constant level after a non-specific early sudden drop/rise, respectively. (C) Fluorescence microscopy images of Jurkat cells incubated with fluorescently tagged cyclotide T20K-CF. Jurkat cells were treated with 10 μM 5/6-carboxy-fluoresceine-tagged cyclotide (green) for 1 h at 37 °C and 5% CO_2_. Before measurements, cell membranes were visualized by a CellMask Orange (red) staining. Scale bars in the upper panel are 50 μm, and those in the lower are 5 μm.

Cellular uptake studies, including the analysis of cytosolic or membrane localization of cyclic cysteine-rich peptides, have been conducted previously with fluorescence-labeled probes and label-free approaches.^47,48^ Fluorescence tagging is powerful in localizing probes in cells as well as for semi-quantitation analysis, but the labeling approach may bias cell uptake.^48^ Here, the total cellular uptake of three peptides (10 μM) in Jurkat cells was measured with label-free quantitation using mass spectrometry: native T20K, the 3SS variant 5 (3SSV5), and the acyclic [Arg|Asn]T20K variant (Figure 4A,B). Native T20K was measured with peptide levels of ∼75 ng (peak) and continuous levels of approximately 50 ng per 200,000 cells within the 2 h experiment. The 3SSV5 as well as the linear T20K variant were only detectable in traces around the lowest level of quantification (85 ng/mL) of the implemented LC–MS method. As a control, we analyzed the cell supernatants to estimate the extracellular concentrations of peptides. In concert with its intracellular rise, native T20K decreased over time in the extracellular solution, but the concentration of the non-native peptide probes remained unchanged. T20K accumulated in cells or associated to the phospholipid membrane, but the label-free methodology would not allow localization of the peptide in cells. Therefore, the cellular uptake and subcellular localization of T20K was analyzed in more detail by fluorescence microscopy using a fluorescence-labeled T20K (T20K-CF, CF-carboxyfluoresceine). This approach demonstrated that T20K-CF is primarily found in the cytosol and is not associated with the membrane (measured by co-staining with membrane marker dyes) (Figure 4C). Labeled T20K-CF accumulated in the cytosol and appeared to be associated with hitherto uncharacterized vesicles (Figure S8A–C) in a time- and concentration-dependent manner (Figure S8D,E). These cellular studies suggest that a linearized loop 6 or a non-native cystine knot configuration can drastically impact on the cytosolic uptake of cyclotides.^48^ Ultimately, alterations to the cysteine network or the cyclic backbone may affect the architecture of the molecule. Therefore, we examined the solution structures of the native T20K cyclotide and the linearized variant to identify molecular features supporting their ability to enter cells and their antiproliferative activity.

### Structural Analysis of the Prototypic Anti-Proliferative Peptide T20K

**Figure 5:**
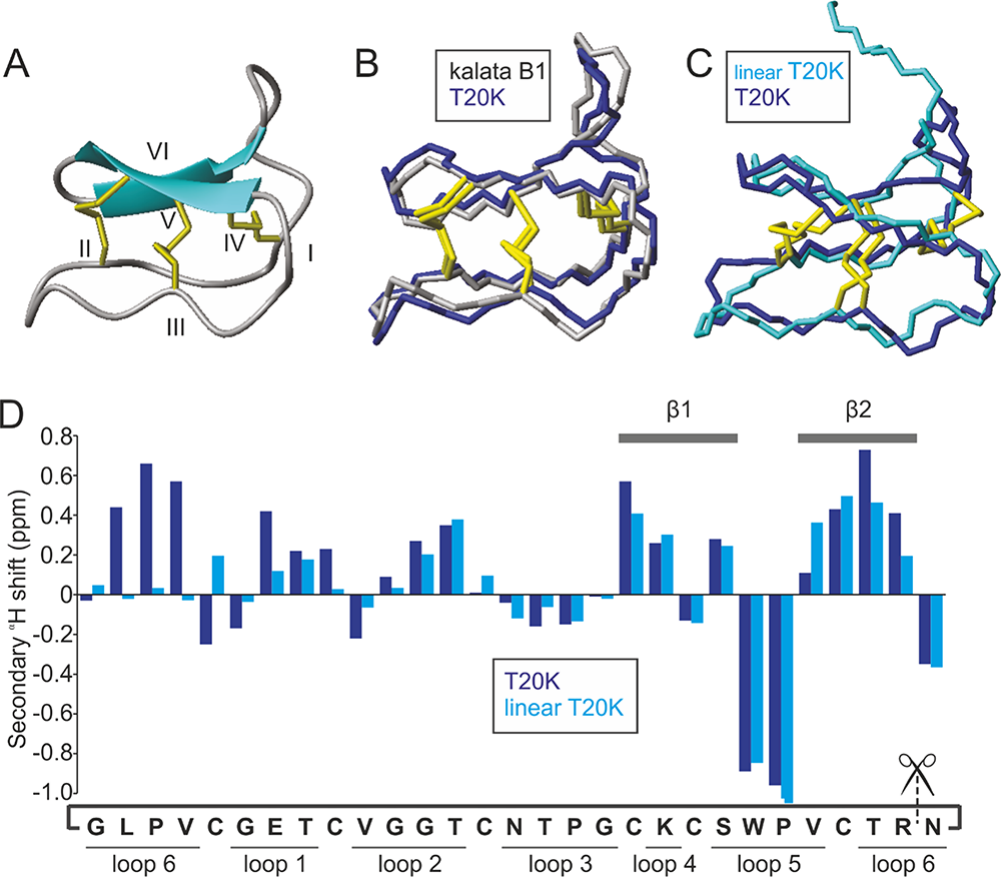
Solution structure of the prototypic antiproliferative cyclotide T20K. (A) Solution structure of T20K as a ribbon diagram. β sheets are represented by blue arrows (showing direction of peptide chain), disulfide bonds in yellow, and cysteine residues numbered I-VI. (B) Backbone structure of T20K (blue) overlaid with that of kalata B1 (grey, PDB: 1nb1). (C) Backbone structure of T20K (blue) overlaid with that of linear T20K (cyan). (D) Secondary αH shift analysis of T20K (blue) and linear T20K (cyan). β strands and intercysteine loops as indicated. The amino acid sequence is written cyclic as per T20K with scissors intersecting the termini of linear T20K.

The secondary structure of T20K in solution was shown previously to be very similar to that of kalata B1, suggesting that the single substitution of threonine for lysine had negligible effect on the backbone conformation.^21^ In this study, the three-dimensional solution structure of T20K was determined by simulated annealing using experimental distance restraints based on NOESY cross-peaks and torsional angle restraints predicted by chemical shift assignments. A summary of the energetic and geometric statistics for a family of the 20 lowest energy structures is given in Table S5. The final structural ensemble overlays with high precision, and the secondary elements are defined by PROMOTIF as two anti-parallel β strands (extending across residues 19 to 22 and 25 to 28) and several β turns, including type I (residues 9–12), type II (residues 16–19), and type VIa1 (residues 22–25). Figure 5 highlights the strong similarity between kalata B1 (PDB: 1nb1) and T20K whose backbone atoms align with a RMSD of 0.78 Å. In contrast, the opening of the cyclic backbone clearly alters the conformation of loop 6 as evidenced by the substantial differences in the secondary αH shifts of residues in this loop compared to those of T20K (Figure 5C). Despite negligible shift differences across the remainder of the sequence, a decrease in the number of long range NOE’s observed across the entire molecule suggests that the structural integrity and tight globular fold normally associated with the CCK motif is absent in linear T20K (Figure S9). In fact, the final structural ensemble of linear T20K displays shorter β strands (involving residues 22 to 23 and 26 to 27) than T20K, although the β turns are similar, for example, type II (residues 17–20) and type VIa1 (residues 23–26). The lengthy N-terminal tail adopts a highly flexible random coil configuration as suggested by the αH shifts and lack of long range NOE’s in this region. Comparison of the backbone atoms of residues 6–29 (of the linear peptide) with those of the equivalent residues of the cyclic molecule reveal that they align with an RMSD of 1.48 Å (Figure 5C).

## Discussion

T20K is a drug candidate for multiple sclerosis that has successfully completed a clinical phase I trial.^20^ It has been developed from the prototypic anti-proliferative cyclotide kalata B1 isolated from *Oldenlandia affinis*. Other cyclotide family members (e.g., from the genus *Palicourea* or *Viola*) are also known to halt the proliferation of T-cells.^11,18,43^ These immunosuppressive cyclotides are characterized by a considerable variation in intercysteine loop sequences but all share the CCK as a highly conserved structural element (Figure S10). Consequently, it was of interest to explore whether, and how, the CCK contributes to the immunosuppressive activity of this peptide family.^11^

Several studies have previously investigated the cystine knot motif with respect to its structural properties and stability,^7,49^ but little is known about the mechanistic details of the knot for bioactivity. We hypothesized that loss of the knot would impact the molecule’s structural integrity and antiproliferative activity toward human PBMCs. Hence, we tested the T-cell anti-proliferative activity of native T20K relative to partial or fully reduced cystine knot variants. Reduction of the three disulfide bonds or Cys modifications with acetamide, *S*-methyl, and *S*-ethyl-maleimide resulted in a full loss of bioactivity in the proliferation assays. Three 1SS as well as three 2SS variants were tested, but they exhibited no activity at concentrations up to 17-fold that of the IC_50_ of native T20K. Clearly, modifying the cystine knot in any way results in a loss of bioactivity. Previous NMR structural studies of the 2SS intermediate of kalata B1, in which the I-IV disulfide bond was either reduced or eliminated by alanine substitution, showed that it had a native-like structure with defined secondary structural motifs.^7^ The structure of T20K has hitherto not been elucidated, but in kalata B1, a 2SS cystine knot reduced variant adopted an overall native-like fold as shown by the comparison of NMR structures.^7,22^ Despite an overall similar sequence of the cystine knot truncated variants, the S-derivatization might also affect physicochemical properties compared to native T20K. The different groups may introduce steric hindrance for a peptide–receptor interaction. To account for such effects a variant with all cysteines replaced with the isostere alanine was prepared for biological evaluation. This T20K-(Cys-to-Ala) variant exhibited no anti-proliferative activity. Therefore, it was necessary to explore in further detail the contribution of the cystine knot and the circular backbone, toward the activity of cyclotides.

The cystine knot has a defined disulfide connectivity (I-IV, II-V, III-VI). *In planta*, a protein disulfide isomerase is thought to catalyze the folding of cyclotides to yield only this highly favored cystine knot configuration.^50^ In contrast, during *in vitro* oxidative folding, conditions are applied that allow the molecules to occupy the lowest energy state, reducing the chance of non-native dead-end 3SS variants or folding intermediates. Kalata-type (i.e., Möbius) but not the bracelet-type cyclotides are routinely amenable for good oxidative folding yields.^51,52^ To examine the contribution of the folding states toward bioactivity, we identified several 3SS variants in folding experiments and four were isolated for assays. Non-native configured 3SS variants did not retain anti-proliferative activity. Despite 15 theoretically possible 3SS variants, only the one configuration that nature has implemented, i.e., the native cystine knot in T20K, exhibited immunosuppressive activity. These observations provide a rationale for the successful evolution of the native cystine knot as the predominant configuration found in many peptides from various origin, for instance, in knottins, hormones, and many other nature-derived peptides.^53,54^

Hence, it was of interest to investigate the bioactive role of the cyclic backbone as the complementary structural element to the cystine knot in cyclotides. The CCK renders cyclotides very stable toward chemical, enzymatic, and thermal degradation,^55^ and later, this remarkable stability was the basis for the successful application of T20K in the experimental autoimmune encephalomyelitis mouse model for multiple sclerosis via oral administration.^19^ Little is known about ADME properties of the drug candidate T20K, but despite its stability, the peptide may eventually become susceptible for proteolytic cleavage and break down to its amino acid building blocks in the body. One biologically relevant site for proteolysis, for instance, via cleavage by the common protease trypsin, is the arginine residue in loop 6. Therefore, a linearized (Arg|Asn) T20K analog was prepared and the folded molecule with native-like disulfide configuration was evaluated as a linearized probe in bioassays and structural studies. Linear T20K was not immunosuppressive anymore, and therefore an intact cyclic loop 6 appears mandatory to define the cystine knot and the molecule antiproliferative activity. These observations are consistent with previous studies where linearized kalata B1 (acyclic permutations) exhibited a loss of function, e.g., for hemolytic activity, compared to the native cyclotide counterpart. Linear variants also occur naturally, with a growing number of such “acyclotides” being reported.^56^ These natural acyclic peptides with sequence homology to cyclotides but with an open loop 6 have been identified in plants from the Violaceae, Fabaceae, Solanaceae, and Rubiaceae and in monocots.^57−59^ Although similar to the native cyclic peptides, subtle changes in chemical or biological properties are inevitable in linear native folded analogues. Acyclotides often have a reduced bioactivity profile, e.g., for hemolytic, anti-HIV, and antimicrobial activities, but this is not a uniform observation, since there are examples of acylcotides with potent antimicrobial (psyle C) or cytotoxic and/or hemolytic activities (chassatide C7, -C8, -C11).^59−61^ A patch of surface-exposed hydrophobic amino acids and a net positive charge guide the membranolytic activity of cyclotides,^27,47,62^ which involves docking to phosphoethanolamine (PE), as described for a range of peptides with intrinsic membrane-activity, including cycloviolacin O2, kalata B1, or hyen D.^37,47,63,64^ In this light, the biological role of natural occurring acyclotides remains elusive. The evolutionary role of a ligated backbone may provide a structural upgrade to the molecule in terms of rigidity. Consequently, hydrophobicity-driven interactions with biological membranes might be different for the linear T20K variant compared to the native peptide (indicated in Figure 1A). This study systematically elaborated the unique role of the CCK motif for the immunosuppressive activity of the drug candidate T20K. Backbone cyclization in many other cystine-knot peptides, such as trypsin inhibitors, conotoxins, or spider toxins, is not mandatory to elicit their bioactivities.^16^

In view of this information, we investigated inactive variants in comparison to T20K in immune cell uptake studies. The total cell uptake of 3SSV5, the linear folded variant, and the native peptide T20K were determined by label-free quantitation. T20K showed fast uptake kinetics, and it was detectable in considerable amounts in Jurkat cells within minutes, but the non-active variants were not detected. The truncated probes likely would be susceptible to proteolytic degradation, e.g., after uptake via the endosomal route into the cell. However, degradation products were not detected in the LC–MS study, which further strengthened our hypothesis that modifications on the cystine knot or the cyclic backbone of T20K affect the molecule’s structure and cell penetrating properties. Fluorescence microcopy experiments further confirmed the cytosolic localization of labeled T20K in immune cells. T20K seems to accumulate in human immune cells quickly to reach substantial intracellular concentrations. Cyclotides have been studied for mammalian cell uptake in the past.^37^ For example, the cyclic Momordica trypsin inhibitor II (MCoTI-II) enters cells via macropinocytosis.^65^ On the other hand, kalata B1 uses both endocytosis mechanisms and direct membrane interaction with PE phospholipids for cellular uptake.^66^ Cystine knot peptides are also internalized via the endosomal route into in early endosomes to subsequently accumulate in lysosomal vesicles.^65,67^ The observed vesicular uptake modality of T20K and the cytosolic location are similar to that described in recent studies.^48,66^ Recently, a cell penetration study using a set of kalata B1 derived probes dissected the total cell uptake into endosomal and cytosolic delivery.^48^ Kalata B1 mutants have substantial capability for endosomal escape, with a high-performing analog reaching cytosolic levels similar to that of the reference cell penetrating peptide TAT-R.^48^

We have demonstrated that the cyclic backbone and the native disulfide connectivity are essential features for the antiproliferative bioactivity of T20K. To further document the differences between active-native and inactive-truncated variants, the in-solution structures of two peptides were elucidated for structural comparison. Although the CCK truncated molecule adopted an overall native-like confirmation, constraints to the long range NOE’s, a limited β sheet formation and the unstructured linear loop were distinctive features. Native T20K has a tight globular fold, whereas its absence in the linear variant led to substantial changes in the molecule’s integrity. An increased flexibility of the acyclotide consequently precludes formation of the hydrophobic patch in loop 6 as well as limiting the ability of linear T20K to interact with and penetrate the cell membrane. Previous comparisons of kalata B1 and acyclic permutants reported similar three-dimensional folds but reduced stability and increased flexibility associated with linearization, which was also reflected by loss of hemolytic activity.^68,69^ It can be speculated that the non-active 3SS variants have similarly deteriorated molecular structures.

In summary, this study examined the roles of the cystine knot, cysteine connectivity, and cyclic backbone to shed light on the role of the CCK motif in bioactivity. Both the native cystine knot and the cyclic backbone were identified as bioactivity guiding elements essential for the antiproliferative activity of T20K on activated human immune cells. The native CCK was essential for cell uptake and cytosolic localization in mammalian cells, while the functional consequence of a distorted CCK was a complete lack of cell-penetrating abilities. The intracellular localization of T20K may therefore be important for its immunosuppressive activity.^48^ The activity of many approved drugs for immune system modulation or suppression is unequivocally linked to their cell-penetrating properties.^44^ Similarly, the CCK motif of cyclotides promotes additional secondary structures and intramolecular hydrogen networks and favors surface presentation of side chain residues of the molecule. Cyclotides, and more specifically the kalata-type family, appear to be superior in penetrating cells and in targeting intracellular receptors than their linear acyclotide counterparts. Therefore, evolution may have driven cyclization not only to create a more stable peptide but to increase the chemical defense diversity of plants and the bioactive repertoire of their weapons (i.e., cyclotides). Thus, backbone cyclization may be recognized as one evolutionary way to upgrade the bioactive properties of a peptide molecule. This study provides further structural and functional understanding of the immunosuppressive properties of cyclotides, such as T20K, that will strengthen their value in peptide drug development. Our work can be motivation to study the structure and mechanism of other naturally circular peptides and their acyclic variants, e.g., RTD-1 or SFTI-I.^70,71^ Cyclotides, as a representative of macrocyclic peptides, together with many other nature-derived cell-penetrating peptides,^72^ are an inspiration for nature-guided chemistry for the development of novel peptide lead molecules and drug candidates.

## Supporting Information Available

Figures and tables demonstrating synthesis and purification of truncated cystine knot variants of T20K (MS spectra, HPLC chromatograms, MS/MS sequencing data); biological experiments and figures of T20K antiproliferation assays as well as time- and concentration-dependent cellular uptake experiments; figures and tables relating to the NMR spectroscopy data of the structural analysis of T20 and variants (PDF)

## Abbreviations

PBMC: peripheral blood mononuclear cells
CCK: cyclic cystine knot
CF: 5–/6-carboxyfluorescein
DTT: dithiothreitol
GSH: glutathione
SPE: solid phase extraction
TFA: trifluoracetic acid
TOF: time-of-flight analyzer
