# Supplementary material for: Importance of the Cyclic Cystine Knot Structural Motif for Immunosuppressive Effects of Cyclotides
Source: ACS Chem Biol. 2021 Sep 30;16(11):2373–86. doi: 10.1021/acschembio.1c00524 (PMC9286316; doi:10.1021/acschembio.1c00524)
Supplement: Supplementary file 1 — cb1c00524_si_001.pdf [file cb1c00524_si_001.pdf]

Supplementary Information

Importance of the cyclic cystine knot structural motif for immunosuppressive effects of cyclotides

Roland Hellinger[#], Edin Muratspahić[#], Seema Devi[$], Johannes Koehbach,[§] Mina Vasileva[#], Peta J. Harvey[§], David J. Craik[§], Carsten Gründemann[&] and Christian W. Gruber*[,#]

[#]Center for Physiology and Pharmacology, Medical University of Vienna, Schwarzspanierstr. 17, 1090 Vienna, Austria

[§]Institute for Molecular Bioscience, Australian Research Council Centre of Excellence for Innovations in Peptide and Protein Science, The University of Queensland, Brisbane, 4072 Queensland, Australia

[$]Institute for Infection Prevention and Hospital Epidemiology, Center for Complementary Medicine, Faculty of Medicine, University of Freiburg, Freiburg, Germany, Breisacher Str. 115B, 79106 Freiburg, Germany

[&]Translational Complementary Medicine, Department of Pharmaceutical Sciences, University of Basel, Klingelbergstr. 80, 4056 Basel, Switzerland

*Correspondence: Christian W. Gruber, christian.w.gruber@meduniwien.ac.at, Tel. +43 40160 31390

Supplementary Figures

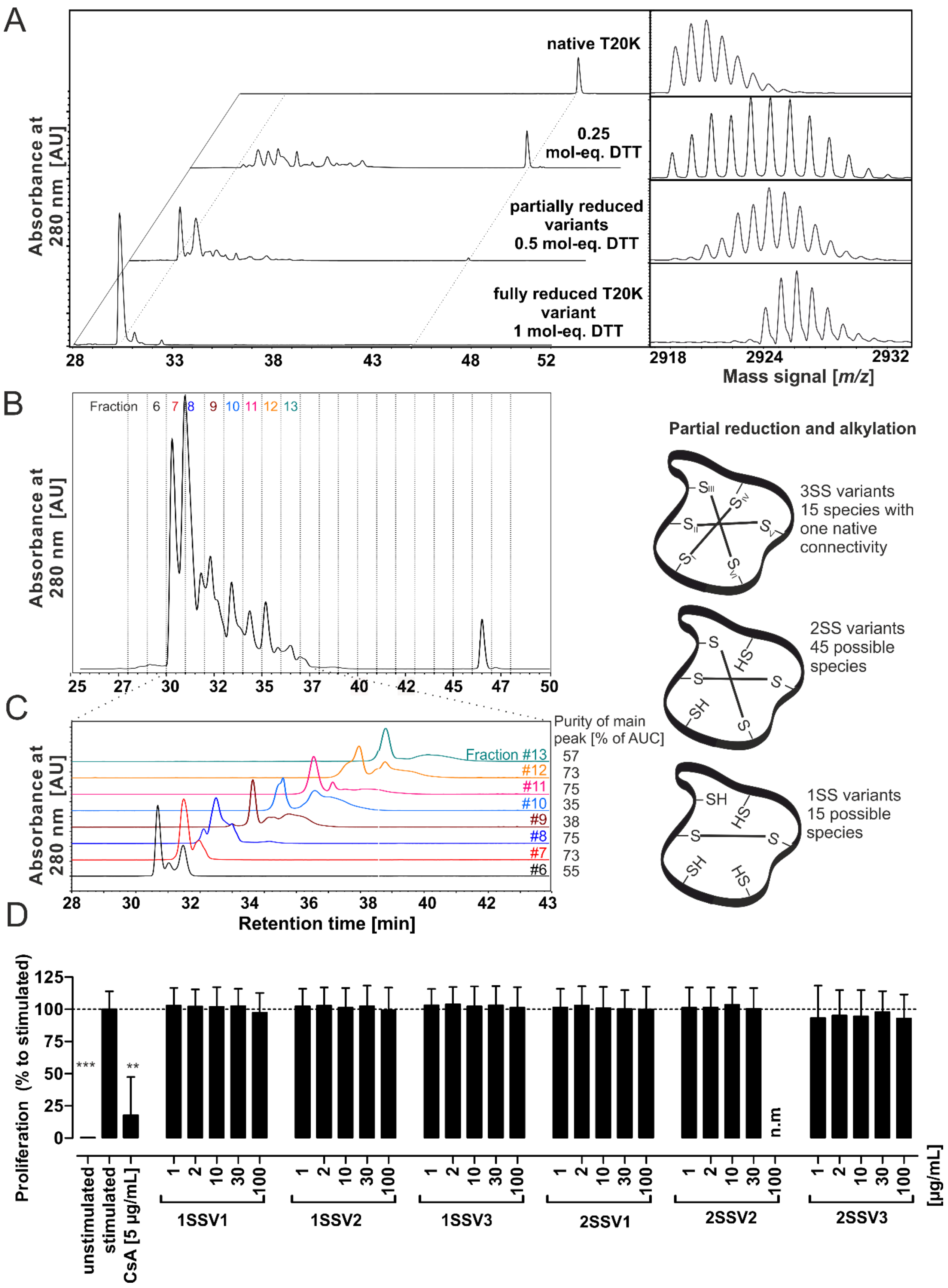

A
native T20K
0.25 mol-eq. DTT
partially reduced variants 0.5 mol-eq. DTT
fully reduced T20K variant 1 mol-eq. DTT
Absorbance at 280 nm [AU]
Mass signal [m/z]
B
Fraction 6 7 8 9 10 11 12 13
Partial reduction and alkylation
3SS variants 15 species with one native connectivity
2SS variants 45 possible species
1SS variants 15 possible species
C
Purity of main peak [% of AUC]
Fraction #13 57
#12 73
#11 75
#10 35
#9 38
#8 75
#7 73
#6 55
Retention time [min]
D
Proliferation (% to stimulated)
unstimulated
stimulated
CsA [5 µg/mL]
1SSV1
1SSV2
1SSV3
2SSV1
2SSV2
2SSV3
n.m
[µg/mL]

Supplementary Figure S1. Preparation of cystine knot deficient 1SS and 2SS variants of T20K. (A) The native folded peptide was used as a starting point to prepare cystine knot truncated 1SS and 2SS variants with native connectivity for the remaining disulfides. A partial reduction of native T20K was obtained with a submolar concentration of reducing agent dithiothreitol compared to the total calculated thiols. The HPLC A_{280} traces for the samples from a time and concentration dependency experiment with reducing agents in 0.0, 0.25-, 0.5- or 1-fold amounts are shown. Submolar concentrations of reducing agent (*e.g.* ratio of 1:0.25) yielded several intermediate species, which was monitored with MALDI-MS (right hand site panel) **(B)** The S-acetamide derivatized peptides were fractionized by HPLC. A chromatographic trace of a semipreparative HPLC experiment with a DiChrom Kromasil C_{18} column (250 × 10 mm, 5 µm) column at flow rate 4 ml/min and a linear gradient of 1% buffer B/min is shown and collected fractions are indicated with dotted lines. The isolated samples showed mass signals for the 1SS, the 2SS variants as well as the T20K-Acm variant. **(C)** HPLC fractions 6-13 were analyzed with Kinetex C_{18} column (150 × 3 mm, 2.6 µm) using a linear gradient as described above with chromatographic resolution superior compared to condition used for fractionation. The analysis revealed co-elution of 1SS or 2SS variants in these fractions. The relative purities, which ranged from 52-78 % for the major peak, were calculated using area under the curve of integrated peaks. A further purification step with better chromatographic resolution (gradient slope was 0.5% mobile phase B / minutes) enabled the isolation of variants with higher purities of each three the 1SS and 2SS variants. **(D)** The variants were evaluated for anti-proliferative activity in the concentrations of 1, 3, 10, 30 and 100 µg/mL. The cystine-knot truncation led to deficient 1SS and 2SS variants with no anti-proliferative activity. All data represent mean ± standard deviation of three biological replicates, expressed relative to stimulated control (= 100%). Asterisks (** $p < 0.01$, *** $p < 0.001$) indicate significant differences compared to stimulated control, (n.m.) indicate not measured.

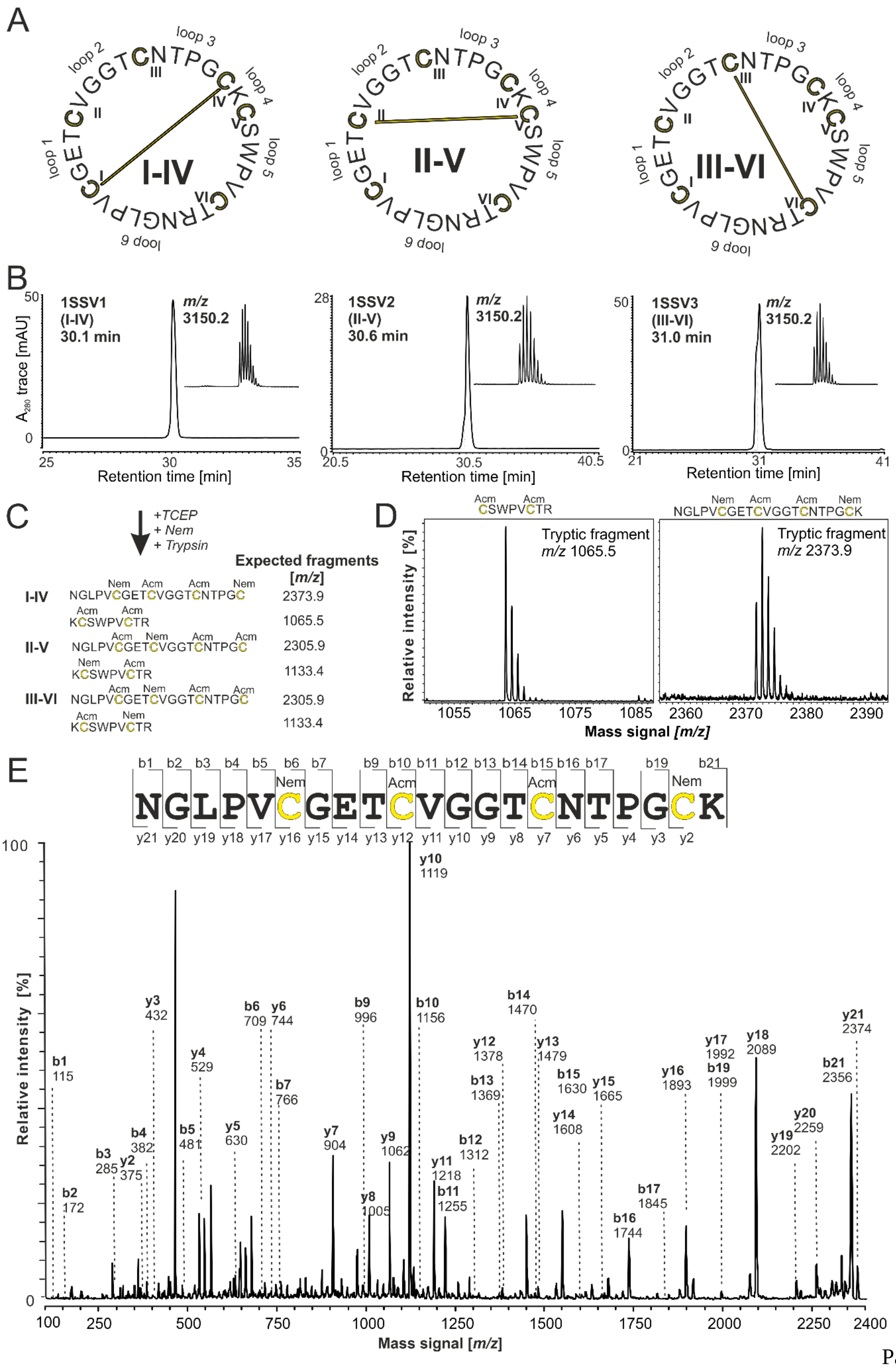
A
I-IV
II-V
III-VI
B
1SSV1 (I-IV) 30.1 min
1SSV2 (II-V) 30.6 min
1SSV3 (III-VI) 31.0 min
m/z 3150.2
A280 trace [mAU]
Retention time [min]
C
+TCEP
+ Nem
+ Trypsin
Expected fragments [m/z]
I-IV NGLPVCGETCVGGTCNTPGC 2373.9
KCSWPVCTR 1065.5
II-V NGLPVCGETCVGGTCNTPGC 2305.9
KCSWPVCTR 1133.4
III-VI NGLPVCGETCVGGTCNTPGC 2305.9
KCSWPVCTR 1133.4
D
Tryptic fragment m/z 1065.5
Tryptic fragment m/z 2373.9
Relative intensity [%]
Mass signal [m/z]
E
NGLPVCGETCVGGTCNTPGCK
Relative intensity [%]
Mass signal [m/z]

Supplementary Figure S2. Isolation and characterization of 1SS variants of T20K. Three 1SS variants of T20K with native connectivity for the remaining disulfide bond were isolated for chemical characterization and bioactivity test. **(A)** An analytical separation with a Kromasil C_{18} column (250 x 4.6, 5 µm) was applied with a separation gradient of 0.5% eluent B/minute to isolate the 1SS variants. Mass spectrometric analysis of the samples obtained *m/z* 3150.11, which corresponded to the theoretical mass signals of S-acetamide stabilized 1SS variants. The compound purities were determined with HPLC analysis at 280 nm. The samples denoted as 1SSV1 had ≥99%, 1SSV2 ≥92 % and 1SSV3 obtained ≥73 % purity **(B)**. The connectivity of the 1SS variants was analyzed by chemical derivatization of cysteines and amino acid analysis. The disulfide bridge was reduced with TCEP in citrate buffer and the sulfhydryl groups were derivatized with N-ethyl maleimide. These mixed alkylated peptides were submitted to tryptic digestion, which obtained two fragments each with specific *m/z* mass signals **(C)**. Because some tryptic fragments were isobaric species, but had cysteine derivatization groups on different sites, MS/MS fragmentation experiments of tryptic fragments were performed, and the disulfide connectivity was determined by *de novo* amino acid sequencing. Exemplarily, the tryptic fragments *m/z* 1065.52 and *m/z* 2373.89 are shown **(D)**. The determined cystine connectivities of 1SS variants were I-IV for 1SSV1 **(E)**, II-V for 1SSV2 III-VI for 1SSV3.

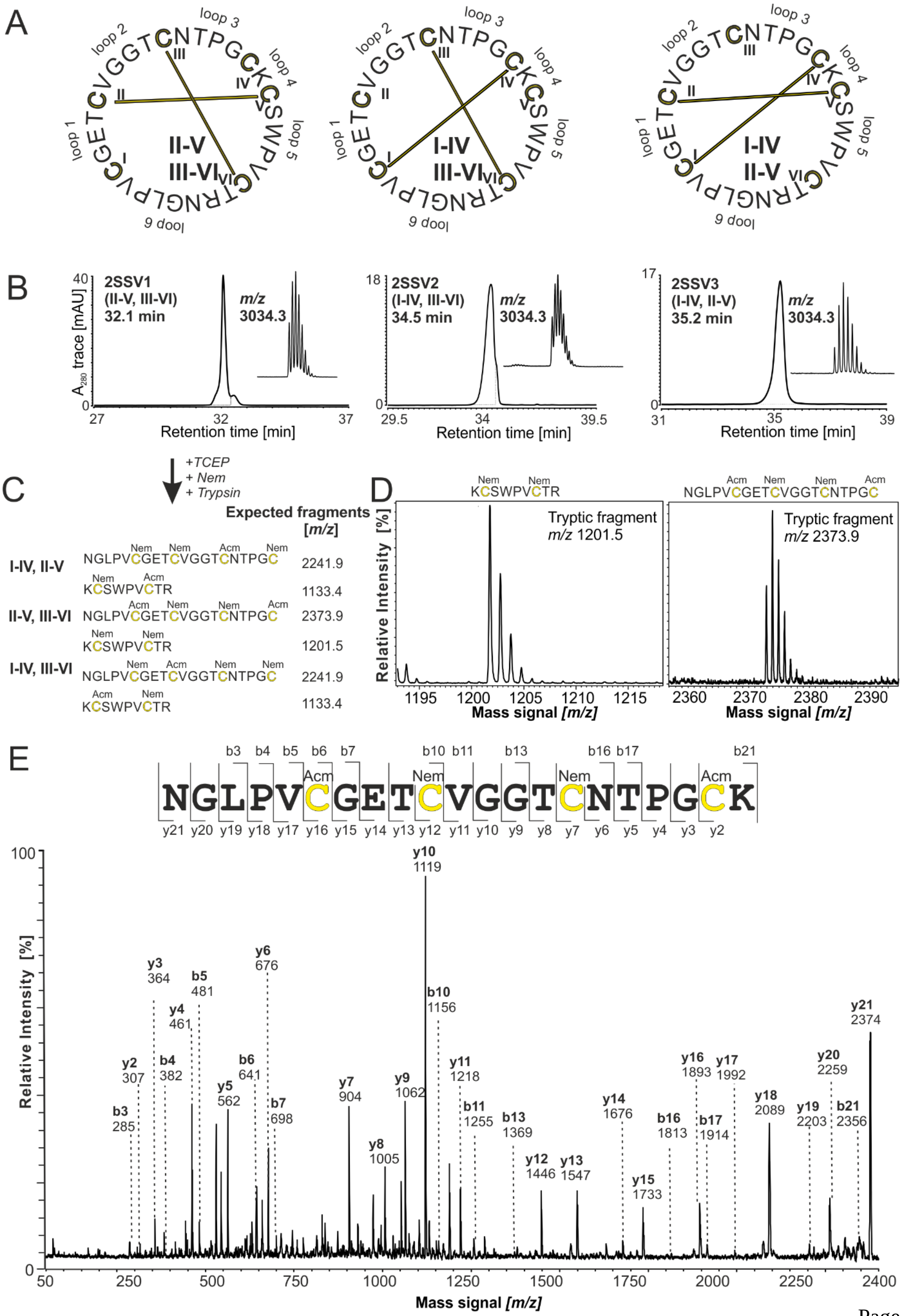
A
loop 1
loop 2
loop 3
loop 4
loop 5
loop 6
II-V
III-VI
I-IV
III-VI
I-IV
II-V
B
2SSV1
(II-V, III-VI)
32.1 min
m/z
3034.3
2SSV2
(I-IV, III-VI)
34.5 min
m/z
3034.3
2SSV3
(I-IV, II-V)
35.2 min
m/z
3034.3
A280 trace [mAU]
Retention time [min]
+TCEP
+ Nem
+ Trypsin
C
Expected fragments [m/z]
I-IV, II-V
NGLPVCGETCVGGTCNTPGC 2241.9
KCSWPVCTR 1133.4
II-V, III-VI
NGLPVCGETCVGGTCNTPGC 2373.9
KCSWPVCTR 1201.5
I-IV, III-VI
NGLPVCGETCVGGTCNTPGC 2241.9
KCSWPVCTR 1133.4
D
KCSWPVCTR
Tryptic fragment
m/z 1201.5
NGLPVCGETCVGGTCNTPGC
Tryptic fragment
m/z 2373.9
Relative Intensity [%]
Mass signal [m/z]
E
NGLPVCGETCVGGTCNTPGCK
Acm
Nem
Nem
Acm
Relative Intensity [%]
Mass signal [m/z]
y10 1119
y21 2374
b10 1156
y3 364
b5 481
y6 676
y4 461
y2 307
b4 382
b6 641
y5 562
b3 285
b7 698
y7 904
y9 1062
y8 1005
y11 1218
b11 1255
b13 1369
y12 1446
y13 1547
y14 1676
y15 1733
b16 1813
y16 1893
y17 1992
b17 1914
y18 2089
y19 2203
y20 2259
b21 2356

Supplementary Figure S3. Isolation and characterization of 2SS variants of T20K. Three 2SS variants of T20K with native connectivity for the remaining disulfide bond were isolated for chemical characterization and bioactivity test **(A)**. Analytical separation with a Kromasil C_{18} column (250 x 4.6, 5 µm) was applied with a separation gradient of 0.5% eluent B/minute to isolate three compounds. Mass spectrometric analysis of the samples obtained *m/z* 3034.10, which corresponded to 2SS variants. The compound purities were determined with HPLC analysis at 280 nm absorption wavelength. The samples denoted as 2SSV1 had ≥ 92%, 2SSV2 ≥ 91% and 2SSV3 obtained ≥ 95% purity **(B)**. The connectivity of the 2SS variants was analyzed by chemical derivatization of cysteines and amino acid analysis. The disulfide bridges were reduced with TCEP in citrate buffer and the sulfhydryl groups were derivatized with N-ethyl-maleimide. These mixed alkylated peptides were submitted to tryptic digestion, which obtained two fragments each with specific *m/z* mass signals **(C)**. Because some tryptic fragments were isobaric species, but have cysteine derivatization groups on different sites, MS/MS fragmentation data of tryptic fragments were prepared, and the disulfide connectivity was determined by *de novo* amino acid sequencing. Exemplarily, the tryptic fragments *m/z* 1201.50 and *m/z* 2373.89 are shown **(D)**, and the determined cystine connectivities of 2SS variants were II-V, III-VI for 2SSV1 **(E)**, I-IV, III-VI for 2SSV2 I-IV, II-V for 2SSV3.

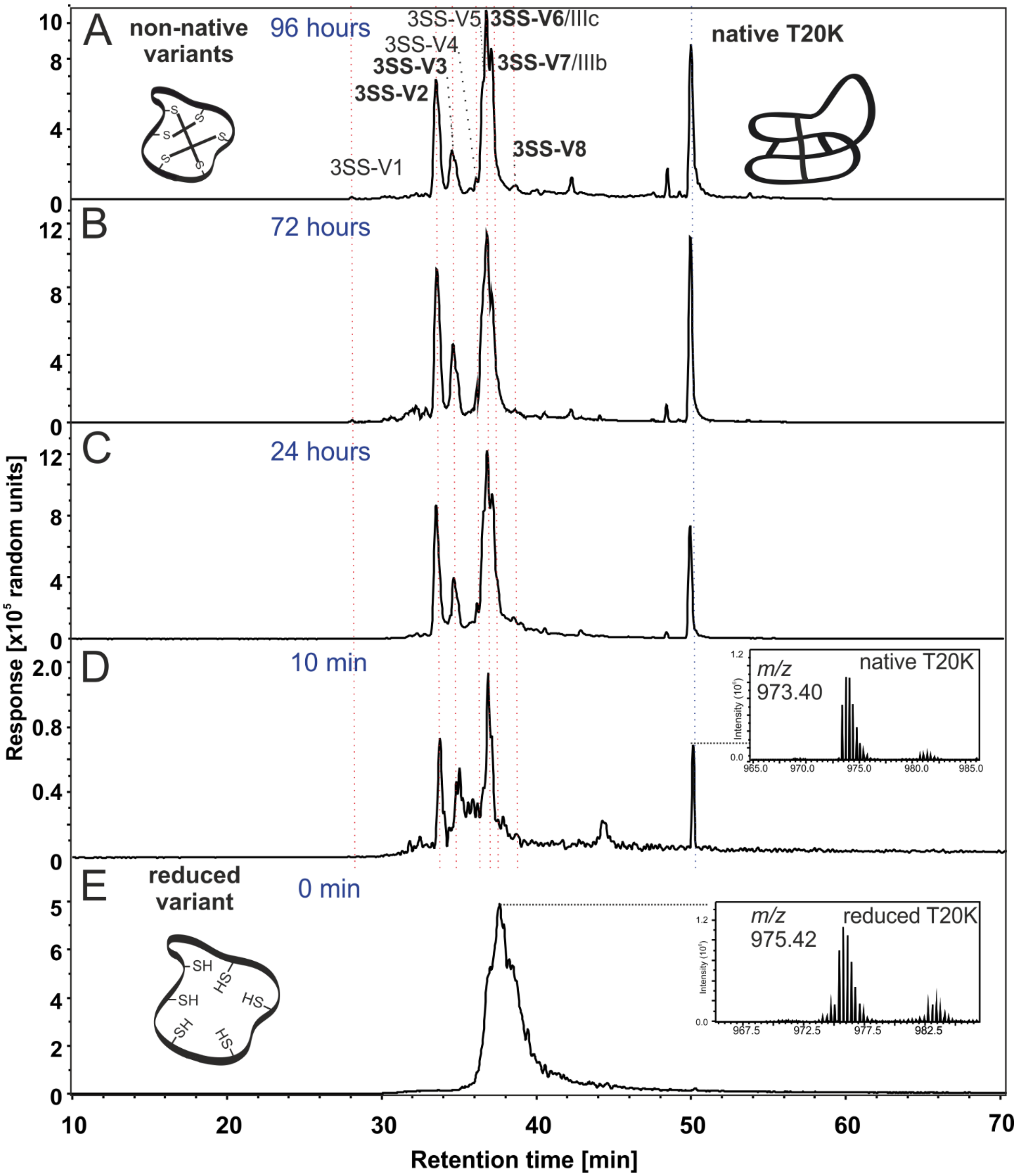

Supplementary Figure S4. Time course analysis of the formation of 3SS variants T20K with LC-MS. Monitoring of the mass trace *m/z* 973.40 (width ±0.02) for the $[M+3H]^{3+}$ ion signal of oxidized 3SS variants was performed to evaluate the oxidative folding. Fully reduced T20K with *m/z* 975.40 was incubated in folding buffer and the formation was analyzed at 0, 10 minutes, 24, 72, 96 hours **(A-E)**. The spectral data were smoothed using a Savitzky Golay function (smoothing width 4.2 seconds and two data points). The non-native 3SS variants and native T20K were generated in folding buffer containing DMSO as oxidative reagent. (See also Table 1 and supplementary Table S3 for chromatographic parameters). The 3SS peptide levels increased over time within the first 24 hours but remained stable in the buffer system for up to 96 hours. Folding intermediates (*e.g.* 1SS with *m/z* 974.76 as well as 2SS with *m/z* 974.08) and dead-end side products were detectable in the time course.

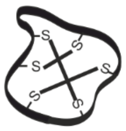

Quality control of non native folded 3SS variants

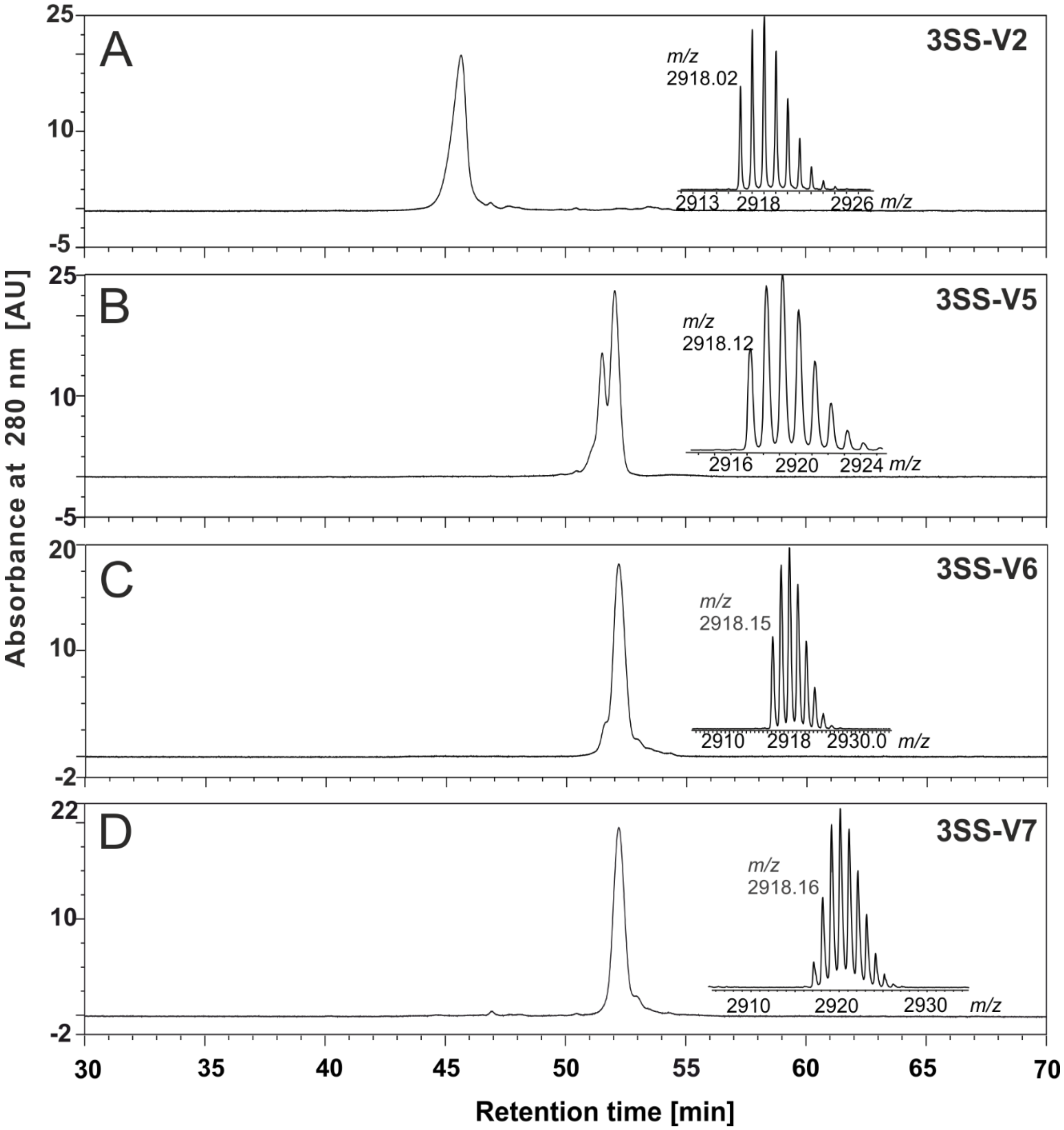

Supplementary Figure S5. Quality control analysis of the isolated 3SS variants. LC-MS enabled the identification of variants, which were labeled with 3SSV1 to -8 according to Supplementary Table S2. **(A-D)** The variants 3SS-V2, -V5, -V6 and -V7 were purified in sufficient amounts (>100 µg) for bioassay. The analytical chromatograms as well as MALDI-MS spectra of these four non-native folded cyclic T20K variants are illustrated to confirm the preparation of the tested peptides.

A

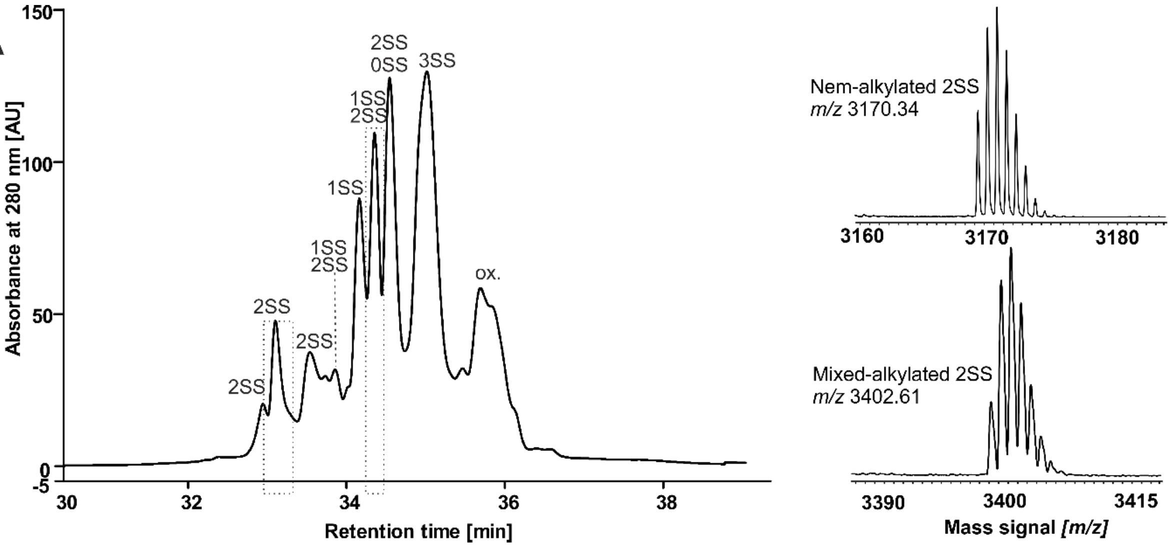

B

Fragment #1 NGLPVCGETCVGGTCNTPGCK — **Fragment #2** CSWPVCTR

**6 10 15 20**	***m/z***	**Expected isobaric configurations**		***m/z***	**1 6**
Acm-Acm-Acm-Acm	2237.9	1	1	1201.5	Nem-Nem
Acm-Acm-Nem-Nem*	2373.9	6	1	1065.5	Acm-Acm
Acm-Acm-Acm-Nem**	2305.9	4	2	1133.5	Acm-Nem***

C

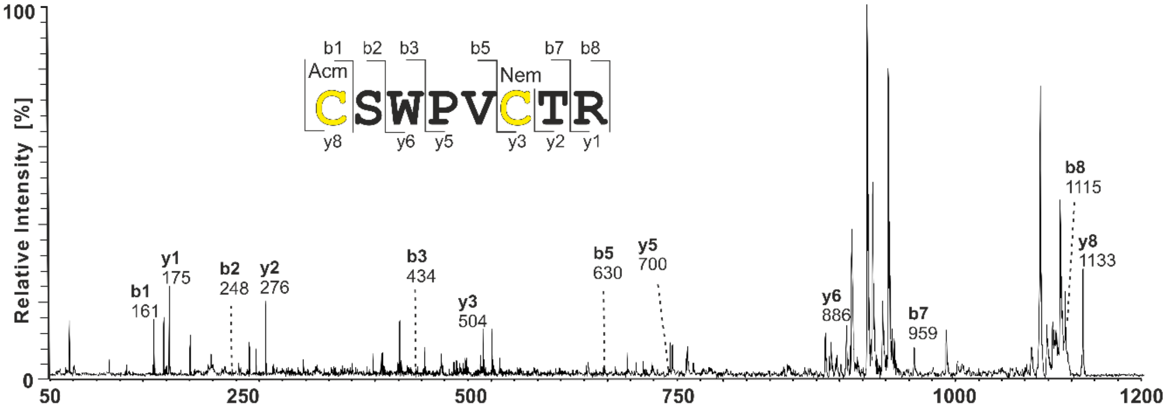

D

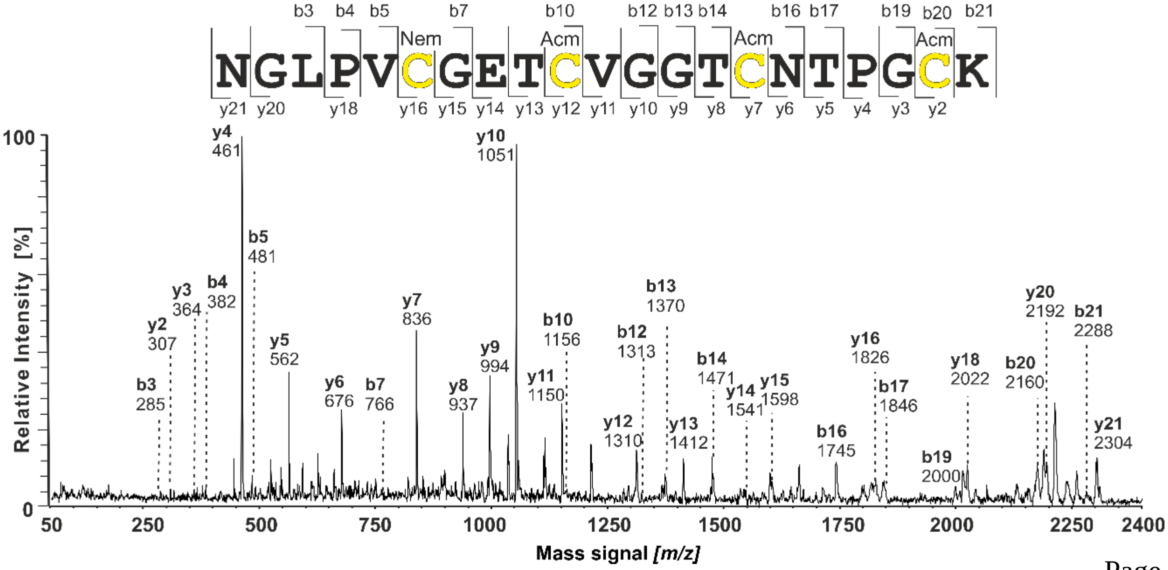

Supplementary Figure S6. Disulfide mapping of 3SS variants of T20K. (A) 3SSV5 material was treated with TCEP (55°C, 3 minutes) to generate partially reduced peptides. The peptides were injected into HPLC for manual peak collection using a Phenomenex Kinetex column with a linear separation gradient of 1% eluent B/min (for details see methods section). The collected peaks were alkylated with N-ethyl-maleimide to prepare Nem-alkylated 1SS and 2SS species. The m/z traces of the samples were acquired and the identified compounds, these were *m/z* 3170.49 for the 2SS, *m/z* 3422.79 for the 1SS, *m/z* 2924.24 for the fully reduced 0SS and *m/z* 2918.19 for the 3SS species, are labeled in the chromatogram. 'Ox.' marks mass signals, which were assigned oxidation side products of the reaction. The two samples, which were used for the assignments of the disulfide connectivity are shown with dotted boxes. Two representative *m/z* traces are shown on the right-hand side for the Nem-alkylated variant (*m/z* 3170.34) and the mixed alkylated 2SS peptide (*m/z* 3402.61). **(B)** Tryptic fragments were prepared from the mixed alkylated 2SS peptides. Considering all possible alkylation patterns for the 2SS analyts the calculated theoretical signature *m/z* mass signals for the proteolytic fragments revealed unique as well as isobaric fragment mass signals. *, ** and *** denote isobaric alkylation patterns, whereof one example out of several theoretical possible configurations is shown. **(C-D)** Representative for the cystine connectivity assignment of all 3SS variants, two annotated MS/MS spectra are provided for the tryptic fragments *m/z* 1133.5 and *m/z* 2305.9 derived from the 3SSV5 species. The Nem-alkylated cysteines were derived from a I-VI disulfide connectivity in this peptide. With the information from two disulfides the third connectivity was obtained by assignment accordingly yielding the full cystine knot configuration of the 3SSV5 peptide (summarized in Supplementary Tables S3 and S4).

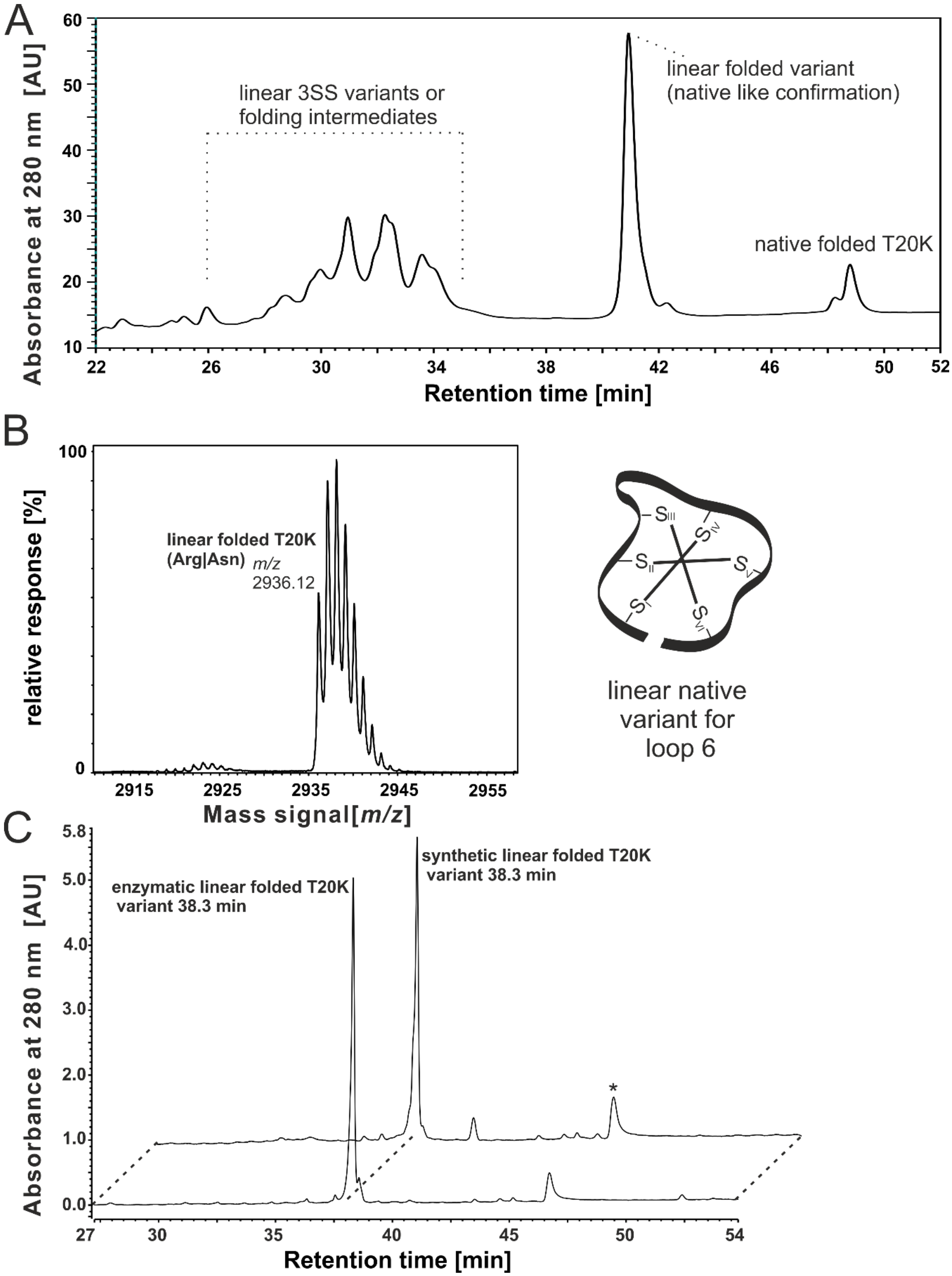
A
Absorbance at 280 nm [AU]
linear 3SS variants or folding intermediates
linear folded variant (native like confirmation)
native folded T20K
Retention time [min]
B
relative response [%]
linear folded T20K (Arg|Asn) m/z 2936.12
Mass signal[m/z]
linear native variant for loop 6
C
Absorbance at 280 nm [AU]
enzymatic linear folded T20K variant 38.3 min
synthetic linear folded T20K variant 38.3 min
*
Retention time [min]

Supplementary Figure S7. Proteolytic preparation and isolation of linear folded variant of T20K. (A) The HPLC chromatogram shows the purification of an oxidative folded linear peptide variant obtained after endoproteinase ArgC proteolysis of the cyclic reduced intermediate peptide. Fully reduced peptide was incubated with the protease and the proteolysis was quenched to avoid complete hydrolysis because a non-specific cleavage at the Lys20 site was detected. The sample was purified with solid phase chromatography and the dried material (containing linear as well as circular peptide) was used for oxidative folding. The peaks for the native, linear folded and several folding intermediates are labeled. The linear folded peptide obtained moderate yields but compared to the non-native folded 3SS variants or folding intermediates, a retention time shift similar as observed for the cyclic folded T20K indicated a native-like confirmation of the peptide. **(B)** MALDI-MS spectra for the ArgC experiment are shown after 24 h oxidative folding. Fully oxidized linear or non-native folded linear 3SS species showed a mass signal of *m/z* 2936.21. **(C)** Synthetic linear 3SS T20K variant and material of this variant derived from enzymatic proteolysis are compared with overlayed HPLC traces (280 nm). A retention time of 38.3 min for both analyst with the standard gradient 1% eluent per minute confirm similar native like fold of the peptides. The tested purity of the materials were > 90% to qualify the peptides for application in antiproliferative activity assays and for structural studies with NMR spectroscopy.

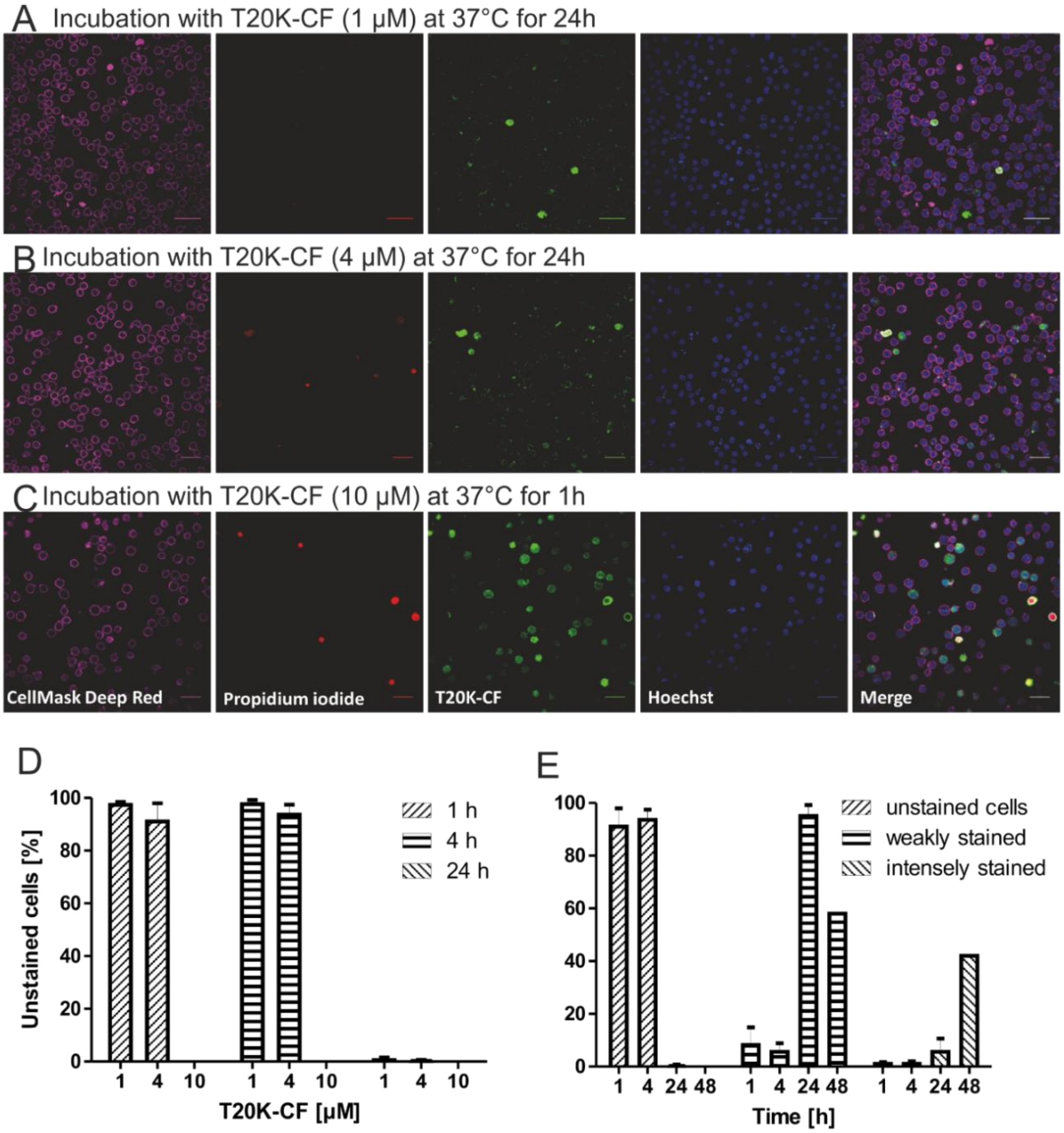

Supplementary Figure S8. Time- and concentration-dependent cellular uptake of fluorescent-labeled T20K in Jurkat cells. (A) Incubation with 1 µM T20K-CF for 24 hours at 37°C, **(B)** incubation with 4 µM T20K-CF for 24 hours at 37°C and **(C)** incubation with 10 µM T20K-CF for 1 h at 37°C. The cellular membrane of Jurkat cells has been stained with 0.5 µg/ml CellMask Deep Red® plasma membrane stain for 5 min at 37°C. The cell nucleus has been visualized, implementing 10 µg/ml Hoechst 33342 for 10 minutes at 37°C. To allow the differentiation between cells with intact membrane and cells with disrupted one, the cells have been incubated for 5 minutes at 37°C with 1 µg/ml propidium iodide. At these concentrations most of the cells have internalized T20K-CF, however an inhomogeneous staining was observed with many cells being moderate and few being heavily stained. Propidium iodide positive cells does co-stain strongly with T20K-CF. The scale bars in the shown images is 50 µm. **(D)** T20K-CF was incubated in 1, 4 and 10 µM on Jurkat cells for 1, 4 or 24 hours and the number of stained cells were counted (expressed as unstained cells in %). The number of fluorescein positive cell increased with the incubation time as well as the concentration of peptide. **(E)** Fluorescein positive cells were categorized in unstained, weakly- as well as intensely stained and the number of cells (expressed as % of unstained) were counted for an incubation with 4 µM T20K-CF for the time points 1, 4, 24 and 48 hours.

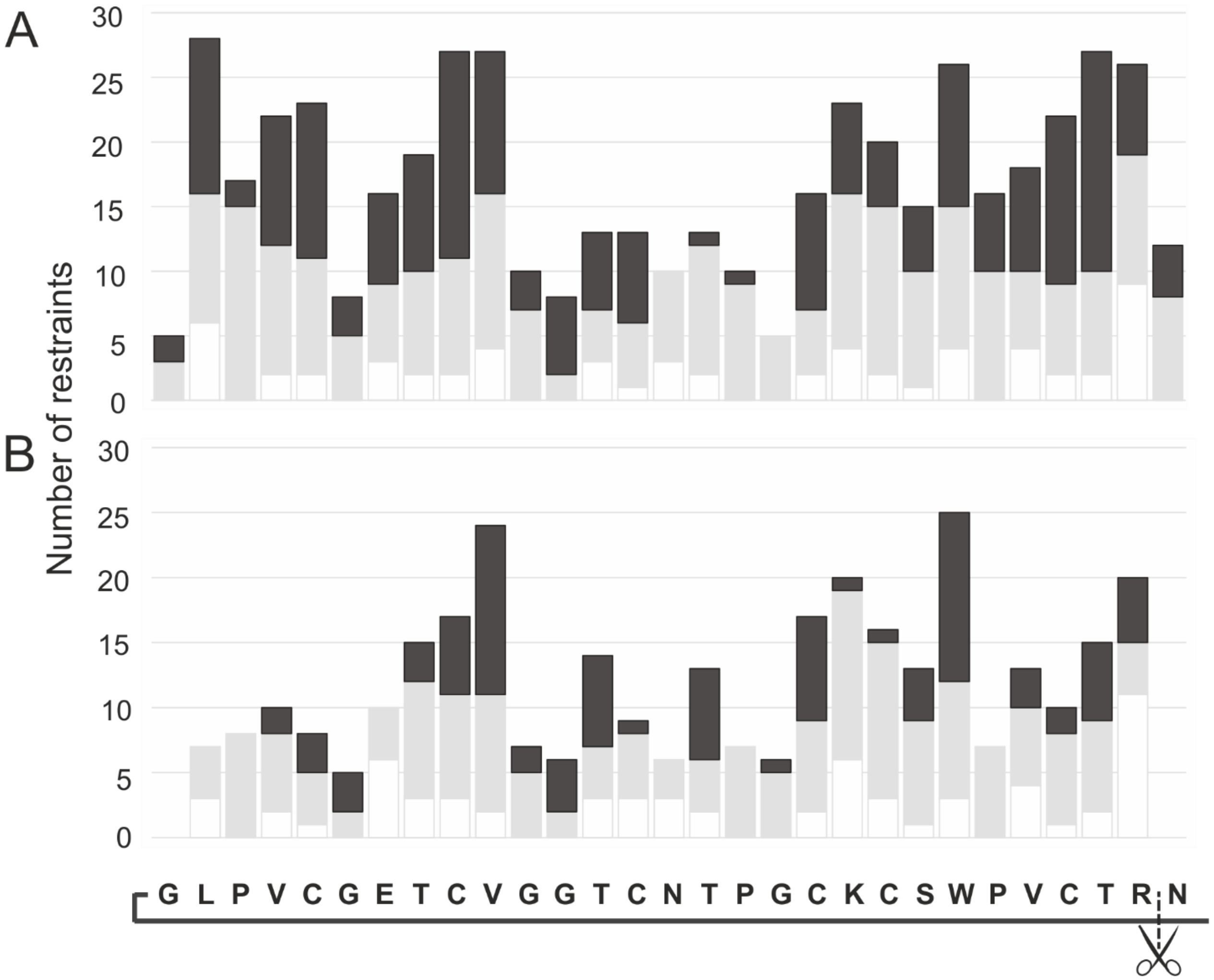

Supplementary Figure S9. Comparison of NOE restraints for the native and linear T20K. Number of NOE restraints determined for T20K (**A**) and linear T20K (**B**). Restraints are distinguished as intra-residue (white), sequential (grey) or mid-long range (black).

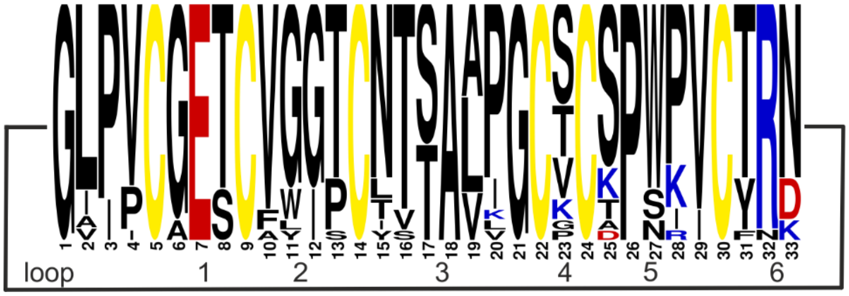

Supplementary Figure S10. Sequence logo of immunosuppressive cyclotides. Amino acid sequences of immunosuppressive cyclotides were used to derive a sequence logo (frequency plot). Cysteines are shown in yellow, acidic residues in red, basic one in blue and all other in black letters. The intercysteine loops are numbered starting with the first N-terminal amino acid from the core region of a generic cyclotide precursor gene.

Supplementary Tables

Supplementary Table S1. Buffer systems for oxidative folding

Buffer system	Salt (final concentration in mM)	pH	Organic modifier	Additives
Standard folding buffer	0.1 M NH_4HCO_3	8.5	50% iPrOH	GSH 4 mM, GSSG 0.5 mM
1	0.1 M NH_4HCO_3	6.0	50% iPrOH	n.a.
2	0.1 M NH_4HCO_3	7.0	50% iPrOH	n.a.
3	0.1 M NH_4HCO_3	8.5	50% iPrOH	n.a.
4	0.1 M NH_4HCO_3	9.0	50% iPrOH	n.a.
5	0.1 M NH_4HCO_3	8.5	50% EtOH	n.a.
6*	0.1 M NH_4HCO_3	8.5	50% DMSO	n.a.
7	0.1 M NH_4HCO_3	8.5	50% iPrOH	500 mM NaCl
8	0.1 M NH_4HCO_3	8.5	50% iPrOH	8 M urea
9	0.1 M NH_4HCO_3	8.5	n.a.	n.a.

n.a., not applicable
*Buffer system 6 was applied to prepare 3SS variants with sufficient yield for peptide isolation

Supplementary Table S2. Isolated and characterized 1SS and 2SS variants of T20K

Peptide variant	Connectivity	Tryptic fragments $[M+H]^+$ [*m/z*]	Retention time [min]	Purity [%] at A_{280}
**1SSV1**	I-IV	2373.90<br>1065.50	30.1	≥99
**1SSV2**	II-V	2305.85<br>1133.42	30.6	≥92
**1SSV3**	III-VI	2305.85<br>1133.42	31.0	≥73
**2SSV1**	II-V, III-VI	2373.60<br>1201.42	32.1	≥92
**2SSV2**	I-IV, III-VI	2441.86<br>1133.42	34.5	≥91
**2SSV3**	I-IV, II-V	2441.86<br>1133.42	35.2	≥95

Supplementary Table S3. Studied 3SS variant peptides of T20K

ID (this study)	Retention time [min]	Relative retention index (this study)	ID/Relative retention index (Gruber et al.)*	Yield [µg[	Purity at 280 nm [%]	Cysteine connectivity	Bioassay
3SSV-1	27.5	1.84	n.a.	n.d.	n.d.	n.d.	not tested
3SSV-2	33.5	0.76	n.a.	257	93.2	I-II, III-IV, V-VI	inactive
3SSV-3	34.5	0.81	n.a.	65	n.d.	n.d.	not tested
3SSV-4	36.2	1.10	n.a.	n.d.	n.d.	n.d.	not tested
3SSV-5	36.7	0.94	n.a.	537	66.0#	I-V, II-VI, III-IV	inactive
3SSV-6	37.0	0.96	IIIc/0.92	837	91.0	I-VI, II-V, III-IV	inactive
3SSV-7	37.5	1.00	IIIb/0.99	473	93.2	I-IV, II-VI, III-V	inactive
3SSV-8	38.6	1.10	n.a.	n.d.	n.d.	n.d.	not tested

n.d., not determined
n.a., not applicable
*relative retention indices were calculated based on retention time of native T20K/kalata B1 and the fully reduced peptides and compared to published 3SS variants (originally identified in Gruber et al.[46])
Sample purity of main peak is 66 %. A second peak (33% AUC) was identified as 3SSV6 by cystine connectivity and retention time.

Supplementary Table S4. Analysis of the cystine connectivity for the 3SS variants of T20K

**3SS variant**	**Partially reduced and Nem-alkylated[1]; Mixed alkylated analyts**<br>**$[M+H]^+$, [*m/z*]**	**Analyzed tryptic fragment[2] and obtained alkylation pattern**<br>**$[M+H]^+$, [*m/z*]**	**Assigned connectivity**	**Full cystine connectivity[3]**
**3SSV2**	*m/z* 3170.49<br>*m/z* 3402. 61<br>= 2SS species	*m/z* 2237.94<br>all Acm<br>*m/z* 1201.52<br>all Nem	V-VI	**I-II, III-IV, V-VI**
	m/z 3170.49<br>*m/z* 3402. 61<br>= 2SS species	*m/z* 2373.99<br>Acm-Acm-Nem-Nem<br>*m/z* 1065.46<br>Acm-Acm	III-IV	
**3SSV5**<br>**Peak 1**	*m/z* 3170.49<br>*m/z* 3402. 61<br>= 2SS species	*m/z* 2305.96<br>Acm- Nem-Acm- Acm<br>*m/z* 1133.49<br>Nem-Acm	II-V	**I-VI, II-V, III-IV**
	m/z 3422.79<br>*m/z* 3538.83<br>1SS species	*m/z* 2373.99<br>Nem-Nem-Acm-Acm<br>*m/z* 1201.52<br>Nem-Nem	III-IV	
**3SSV5**<br>**Peak 2**	*m/z* 3170.49<br>*m/z* 3402. 61<br>= 2SS species	*m/z* 2305.96<br>Acm- Nem-Acm- Acm<br>*m/z* 1133.49<br>Acm-Nem	II-VI	**I-V, II-VI, III-IV[4]**
	m/z 3170.49<br>*m/z* 3402. 61<br>= 2SS species	*m/z* 2305.96<br>Nem-Acm-Acm-Acm<br>*m/z* 1133.48<br>Nem-Acm	I-V	
**3SSV6**	*m/z* 3170.49<br>*m/z* 3402. 61<br>= 2SS species	*m/z* 2305.96<br>Nem-Acm-Acm-Acm<br>*m/z* 1133.48<br>Acm-Nem	I-VI	**I-VI, II-V, III-IV**
	m/z 3170.49<br>*m/z* 3402. 61<br>= 2SS species	*m/z* 2305.96<br>Acm-Nem-Acm-Acm<br>*m/z* 1133.48<br>Nem-Acm	II-V	
**3SSV7**	*m/z* 3170.49<br>*m/z* 3402. 61<br>= 2SS species	*m/z* 2373.99<br>Nem-Acm-Acm-Nem<br>*m/z* 1065.46<br>Acm-Acm	I-IV	**I-IV, II-VI, III-V**
	m/z 3170.49<br>*m/z* 3402.61<br>= 2SS species	*m/z* 2305.96<br>Acm-Acm-Nem-Acm<br>*m/z* 1133.49<br>Nem-Acm	III-V	

[1] Several partially reduced and N-ethyl-maleimide alkylated species were isolated with HPLC, afterwards the peptide was fully reduced as well as alkylated with iodoacetamide and this mixed alkylated peptide was digested with trypsin. Tryptic fragments were used for MS/MS fragmentation experiments.
[2] Two theoretical tryptic fragments are expected from T20K: #1 NGLPVCGETCVGGTCNTPGCK with cysteines at position 6-10-15-20 and #2 CSWPVCTR with cysteines at position 1-6.
[3] The experimental assignment of two cystines within the 3SS variant allows the rational full assignment of the cystine connectivity; the native cystine connectivity of T20K is I-IV, II-V, III-VI
[4] 3SSV5 Peak 2 is identical with 3SSV6

Supplementary Table S5. Statistical analysis of T20K and linear T20K structures[a]

**Experimental restraints**	**T20K**	**linear T20K**
total no. distance restraints	278	198
intraresidue	60	65
sequential	116	86
medium range, *i*-*j*<5	41	25
long range, *i*-*j*≥5	61	22
hydrogen bond restraints	14	6
dihedral angle restraints		
phi	18	17
psi	14	7
chi1	7	3
**Deviations from idealized geometry**		
bond lengths (Å)	0.011 ± 0.000	0.010 ± 0.001
bond angles (deg)	1.013 ± 0.049	1.060 ± 0.056
impropers (deg)	1.58 ± 0.15	1.64 ± 0.12
NOE (Å)	0.008 ± 0.002	0.012 ± 0.002
cDih (deg)	0.180 ± 0.093	0.146 ± 0.113
**Mean energies (kcal/mol)**		
overall	-911 ± 30	-839 ± 43
bonds	10.7 ± 0.8	10.3 ± 1.2
angles	28.1 ± 2.7	30.3 ± 3.5
improper	15.2 ± 2.4	16.3 ± 2.0
van Der Waals	-106.7 ± 2.8	-91.3 ± 4.8
NOE	0.02 ± 0.01	0.03 ± 0.01
cDih	0.20 ± 0.21	0.11 ± 0.14
electrostatic	-977 ± 33	-919 ± 43
**Violations**		
NOE violations exceeding 0.2 Å	0	0
Dihedral violations exceeding 2.0 Å	0	0
Rms deviation from mean structure, Å		
backbone atoms	0.62 ± 0.19	0.81 ± 0.19[b]
all heavy atoms	1.19 ± 0.21	1.62 ± 0.37[b]
**Stereochemical quality**[c]		
Residues in most favoured Ramachandran region, %	81.9 ± 7.3	85.7 ± 5.3
Ramachandran outliers, %	0 ± 0	1.3 ± 1.8
Unfavourable sidechain rotamers, %	0.0 ± 0.0	0.0 ± 0.0
Clashscore, all atoms	6.4 ± 3.0	8.0 ± 3.7
Overall MolProbity score	1.9 ± 0.2	2.0 ± 0.2

[a]All statistics are given as mean ± SD.
[b] Rms deviations for linear T20K are calculated for residues 6-29 only.
[c] According to MolProbity published in Chen, V.B. et al. 2010[42]

Bibliography (please refer to manuscript for numbering)

42. Chen, V. B., Arendall, W. B., 3rd, Headd, J. J., Keedy, D. A., Immormino, R. M., Kapral, G. J., Murray, L. W., Richardson, J. S., and Richardson, D. C. (2010) MolProbity: all-atom structure validation for macromolecular crystallography, *Acta Crystallogr D Biol Crystallogr 66*, 12-21.
46. Gruber, C. W., Cemazar, M., Clark, R. J., Horibe, T., Renda, R. F., Anderson, M. A., and Craik, D. J. (2007) A novel plant protein-disulfide isomerase involved in the oxidative folding of cystine knot defense proteins, *J Biol Chem 282*, 20435-20446.
